# Temporal Changes in Vaginal Microbiota and Genital Tract Cytokines Among South African Women Treated for Bacterial Vaginosis

**DOI:** 10.3389/fimmu.2021.730986

**Published:** 2021-09-14

**Authors:** Andile Mtshali, James Emmanuel San, Farzana Osman, Nigel Garrett, Christina Balle, Jennifer Giandhari, Harris Onywera, Khanyisile Mngomezulu, Gugulethu Mzobe, Tulio de Oliveira, Anne Rompalo, Adrian Mindel, Salim S. Abdool Karim, Jacques Ravel, Jo-Ann S. Passmore, Quarraisha Abdool Karim, Heather B. Jaspan, Lenine J. P. Liebenberg, Sinaye Ngcapu

**Affiliations:** ^1^Centre for the AIDS Programme of Research in South Africa (CAPRISA), Durban, South Africa; ^2^Department of Medical Microbiology, University of KwaZulu-Natal, Durban, South Africa; ^3^KwaZulu-Natal Research Innovation and Sequencing Platform, Nelson R Mandela School of Medicine, University of KwaZulu-Natal, Durban, South Africa; ^4^Discipline of Public Health Medicine, University of KwaZulu-Natal, Durban, South Africa; ^5^Institute of Infectious Disease and Molecular Medicine (IDM), University of Cape Town, Cape Town, South Africa; ^6^Department of Gynecology and Obstetrics, Johns Hopkins University, Baltimore, MD, United States; ^7^Department of Epidemiology, Columbia University, New York City, NY, United States; ^8^Department of Microbiology and Immunology, University of Maryland School of Medicine, Baltimore, MD, United States; ^9^Department of Epidemiology and Public Health, University of Maryland School of Medicine, Baltimore, MD, United States; ^10^Department of Medical Virology, National Health Laboratory Service, Cape Town, South Africa; ^11^Seattle Children’s Research Institute, University of Washington Department of Pediatrics and Global Health, Seattle, WA, United States

**Keywords:** vaginal microbiota, genital tract cytokines, bacterial vaginosis, metronidazole treatment, BV recurrence microbial and cytokine profiles by BV treatment

## Abstract

The standard treatment for bacterial vaginosis (BV) with oral metronidazole is often ineffective, and recurrence rates are high among African women. BV-associated anaerobes are closely associated with genital inflammation and HIV risk, which underscores the importance of understanding the interplay between vaginal microbiota and genital inflammation in response to treatment. In this cohort study, we therefore *investigated *the effects of metronidazole treatment on the vaginal microbiota and genital cytokines among symptomatic South African women with BV [defined as Nugent score (NS) ≥4] using 16S rRNA gene sequencing and multiplex bead arrays. Among 56 BV-positive women, we observed short-term BV clearance (NS <4) in a proportion of women six weeks after metronidazole treatment, with more than half of these experiencing recurrence by 12 weeks post-treatment. BV treatment temporarily reduced the relative abundance of BV-associated anaerobes (particularly* Gardnerella vaginalis *and* Atopobium vaginae*) and increased lactobacilli species (mainly *L. iners*), resulting in significantly altered mucosal immune milieu over time. In a linear mixed model, the median concentrations of pro-inflammatory cytokines and chemokines were significantly reduced in women who cleared BV compared to pre-treatment. BV persistence and recurrence were strongly associated with mucosal cytokine profiles that may increase the risk of HIV acquisition. Concentrations of these cytokines were differentially regulated by changes in the relative abundance of BVAB1 and* G. vaginalis*. We conclude that metronidazole for the treatment of BV induced short-term shifts in the vaginal microbiota and mucosal cytokines, while treatment failures promoted persistent elevation of pro-inflammatory cytokine concentrations in the genital tract. These data suggest the need to improve clinical management of BV to minimize BV related reproductive risk factors.

## Introduction

Vaginal microbiota dominated by *Lactobacillus* species are considered optimal for the female genital tract (FGT), because they regulate host immunity and inhibit pathogen colonization by producing antimicrobial substances, like lactic acid and bacteriocins (1, 2). However, this paradigm of an optimal vaginal microbiota may not apply as clearly to women of African descent, because high proportions of non-*Lactobacillus*-dominant microbiota have frequently been found in asymptomatic women (3). *Culture-independent 16S rRNA gene sequencing* studies have shown that the majority of young, healthy, black South African women have vaginal microbiota with low *Lactobacillus* abundance and high diversity (4–6). These types of microbiota are reminiscent of those found associated with the condition known as bacterial vaginosis (BV), which is also characterized by a lack of *Lactobacillus* species, high pH and can present clinically with vaginal discharge, a fish-like odor, vaginal discomfort and urinary symptoms (7). BV is common in women of African descent and characterized by the colonization with a diverse spectrum of primarily anaerobic bacteria (7), elevated genital inflammation and thus increased HIV risk (4–6). Several studies have demonstrated that in the vagina, an increased bacterial diversity or a shift to higher quantities of specific bacterial species correlates with genital inflammation. Specifically, non-*Lactobacillus* species and BV-associated anaerobes enhanced pro-inflammatory cytokines and chemokines production (8, 9). *In vitro* studies have further shown that the relative abundance of *Prevotella bivia* was closely associated with increased levels of vaginal pro-inflammatory cytokines and chemokines (4, 10).

Despite not being considered an infection, per se, symptomatic BV is usually treated with either oral or topical metronidazole or clindamycin (11). However, antibiotic treatment of BV is often ineffective, and recurrence rates following treatment are high (12). After antibiotic treatment of BV, several studies have shown a modest reduction in bacterial counts of BV-associated microorganisms, which was inversely associated with increased relative abundances of *Lactobacillus* species (13–15). Verwijs et al. (2019) showed that only 16% of Rwandan women experienced a reduction of ≥50% in mean concentrations of BV-associated bacteria after metronidazole treatment. In Kenyan women, BV treatment was successful in reducing recurrence of BV by 10% (16), and a 36% increase in the proportion of women with a *Lactobacillus*-dominant vaginal microbiota (17), but this was short-lived after stopping oral metronidazole (18). The use of probiotic *L. crispatus* after treatment (Lactin-V) with vaginal metronidazole resulted in a significantly lower incidence of BV recurrence *versus* placebo at 12 weeks (19).

Given the association of both diverse vaginal communities and inflammation with poor reproductive health outcomes, a better understanding of the impact of current BV treatment on the vaginal microbial environment and genital inflammation is needed. In this cohort study among young South African women, *we set out to investigate the* longitudinal impact of standard BV treatment on the vaginal microbiota. We hypothesized that metronidazole treatment would reduce the abundances and diversity of vaginal bacteria, as well as genital inflammation, and that women with persistent or recurrent BV would have distinctly different mucosal cytokine and chemokine concentration compared to women who cleared the BV.

## Materials and Methods

### Study Design, Participants, and Sample Collection

**Figure 1:**
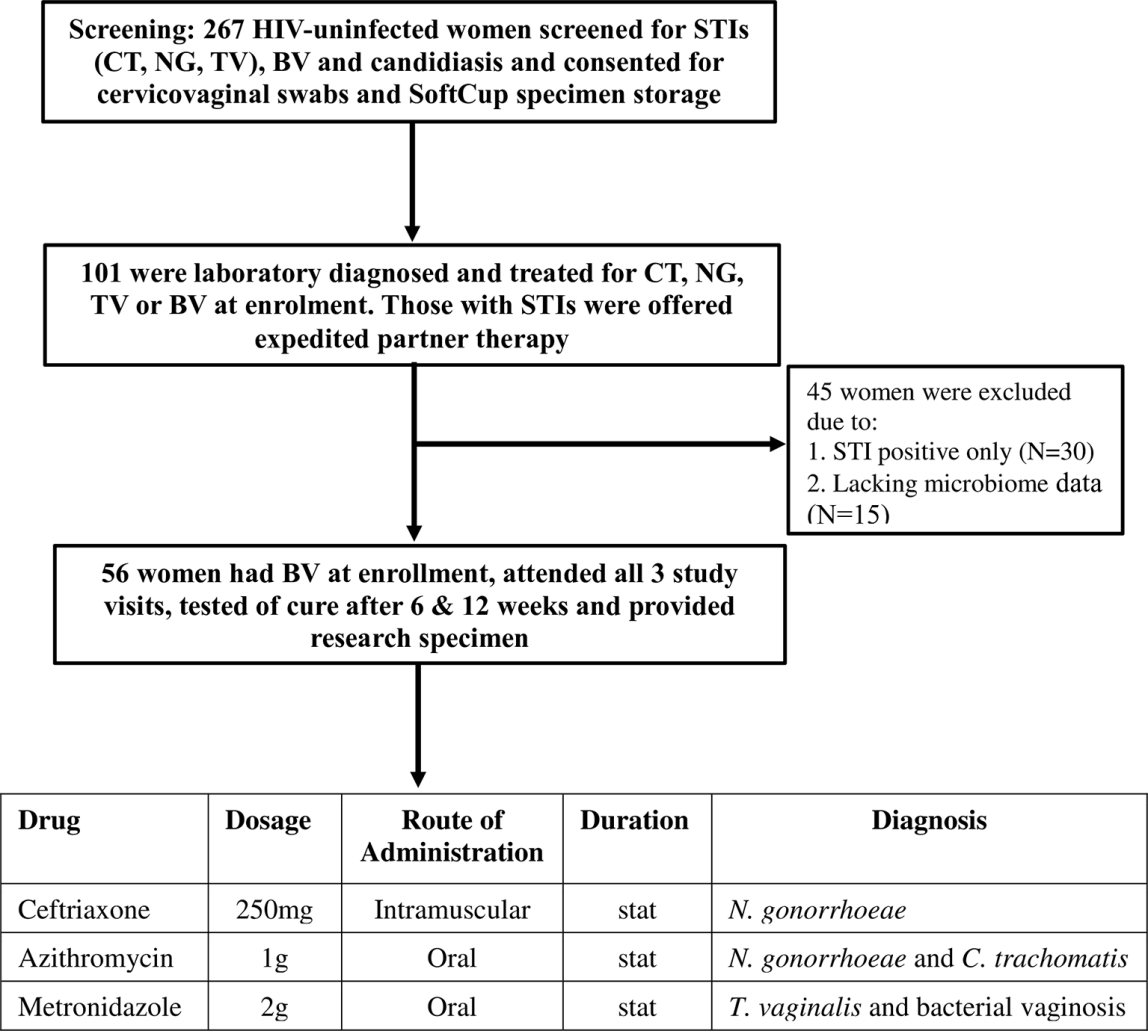
Screening, enrolment and follow-up of the study participants in the CAPRISA 083 cohort study. Consenting women underwent bacterial vaginosis (BV) and Candida screening and point-of-care (POC) testing for sexually transmitted infections (STI): chlamydia (CT), gonorrhoea (NG) or Trichomoniasis (TV). Women diagnosed with Nugent-BV and any STI were treated, tested for cure at follow-up visits (6 and 12 weeks) and offered expedited partner therapy to deliver to their partners. Mucosal samples were collected at all visits. Women diagnosed with STIs only and/or who did not have vaginal microbiome data were excluded from the study and only those who attended all 3 visits and provided specimens were included.

The CAPRISA 083 study screened 267 HIV-uninfected women, and enrolled 101 women with a laboratory-diagnosed STI and/or BV-intermediate or positive into a cohort (flow diagram in **Figure 1**) (20). Women who were pregnant or who had received antibiotic treatment within seven days of sampling were excluded from the study. STI screening was conducted at the clinic by GeneXpert (Cepheid, CA, USA) and by standard PCR multiplex assay (21) was performed. General physical examination, followed by a speculum examination to assess for vulval, vaginal and cervical abnormalities was also performed at each visit. Participants diagnosed with *Chlamydia trachomatis* (azithromycin 1g oral)*, Neisseria gonorrhoeae* (ceftriaxone 250mg intramuscular and azithromycin 1g oral)*, Trichomonas vaginalis* (metronidazole 2g oral), symptomatic BV with Nugent score ≥4 (metronidazole 2g oral single dose) or candidiasis (clotrimazole 500mg pessary and clotrimazole 1% cream) were treated with regimens recommended in the South African STI treatment guidelines (22). At follow-up visit, women who remained BV positive after initial treatment were further offered metronidazole 400mg for 5 days. Enrolled and treated women were asked to return after 6 weeks and 12 weeks for follow-up examinations, further collections of specimens and retesting for STIs, Nugent-BV and candidiasis. Prostate-specific antigen (PSA) was measured in SoftCup supernatants by ELISA (R&D Systems, MN, USA) to account for the effects of recent sexual activity on measured parameters. The protocol for this study was approved by the Ethics Review Committee of the University of KwaZulu-Natal (BREC number: BE403/16).

### BV Classification Using Nugent Score Across Visits

A vaginal swab was used for Gram staining for the diagnosis of candidiasis and BV using Nugent’s criteria (a score of 0 - 3 was considered BV negative, 4 - 6 intermediate BV, and 7 - 10 BV positive) (23). Nugent-BV in this analysis was defined as Nugent score ≥4 (13, 24). BV groups were then classified into women who “cleared”, those who “persisted”, and “recurrent” BV. The term “cleared” was defined as having a Nugent score ≥4 at baseline and 6 weeks follow-up visits, but <4 at the 12 weeks follow-up visit. The term “persistence” was defined as Nugent Score ≥4 at baseline visit and remain so at the next consecutive visits. The term “recurrent” was defined as Nugent score <4 at 6 weeks follow-up visit, but Nugent score increase to >4 at the next visit.

### Bacterial DNA Extraction

Bacterial DNA was extracted from the genital swabs for 16S rRNA gene sequencing using the PowerSoil DNA kit (MoBio, CA, USA), after mechanical and enzymatic disruption using 5μl of lysozyme at 10 mg/ml (Sigma-Aldrich, MO, USA); 13μl of mutanolysin at 11,700 U/ml (Sigma-Aldrich, MO, USA) and 3.2 μl of lysostaphin a 1mg/ml (Sigma-Aldrich, St. Louis, MO) and was incubated at 37°C for 60 min. Mechanical disruption was then performed using a Precellys 24 tissue homogenizer (Bertin instruments, Montigny-le-Bretonneux, France) at 3x30s at speed setting m/s 5.5 (equivalent to 5500rpm). DNA was then purified from the using the Agencourt AMPure XP (Beckman Coulter, CA, USA). DNA was quantified using Qubit 2.0 Fluorometer (Invitrogen, MA, USA).

### Characterization of the Vaginal Microbiota

The V3-V4 hypervariable region was amplified using universal 319F/806R primers, and purified libraries consisting of ~120 pooled samples were sequenced on the Illumina MiSeq platform (paired-end sequence reads with v3 chemistry). FastQC was used to perform data quality control of raw sequences and to infer parameters for upstream processing. Divisive Amplicon Denoising Algorithm (DADA) 2 was used to infer amplicon sequence variants (ASVs) (25). The SILVA ribosomal RNA database (25) was used for taxonomic assignment. The ASVs for key genera such as *Lactobacillus, Prevotella, Sneathia, Mobiluncus*, were further refined with speciateIT (version 1.0, http://ravel-lab.org/speciateIT) to the species level. The resulting phylogenetic tree, ASV table, and taxonomic table combined with relevant metadata were consolidated into a phyloseq object using the phyloseq R package (26). Community state types were inferred using VALENCIA (VAginaL community state typE Nearest CentroId clAssifier) (27), a nearest centroid classification method for vaginal microbial communities based on composition, implemented in python (version 3.6) and has the pandas module as a dependency (28). VALENCIA uses distance matrices to classify similarity of bacterial community structures between individual samples based on species proportions to each reference centroid were calculated using the Yue-Clayton θ (29).

### Measurement of Cytokine Biomarkers of Genital Inflammation

Cervicovaginal SoftCup supernatants collected at matching time points with vaginal swabs were used to measure concentrations of 48 cytokines by Bio-Plex Pro-Human Cytokine Group I (27-Plex Panel) and Group II (21-Plex Panel) kits (Bio-Rad Laboratories, USA) kits as previously reported (30). The cytokine panel included chemokines, pro-inflammatory cytokines, adaptive, growth factors and anti-inflammatory: Interleukin (IL)-1β, IL-1Rα, IL-2, IL-4, IL-5, IL-6, IL-7, IL-8, IL-9, IL-10, IL-12p70, IL-12p40, IL-16, IL-18, IL-1A, IL-2RA, IL-3, IL-13, IL-15, IL-17, basic fibroblast growth factor (FGF-basic), cutaneous T-cell attracting chemokine (CTACK), Eotaxin, granulocyte colony-stimulating factor (G-CSF), GM–CSF,GRO-α, hepatocyte growth factor (HGF), Interferon (IFN)-γ, IFN-α2, interferon-γ -inducible protein (IP)-10, leukemia inhibitory factor (LIF), monocyte chemoattractant protein (MCP)-1, MCP-3, macrophage colony-stimulating factor (M-CSF), monokine induced by gamma- interferon (MIG), Macrophage migration inhibitory factor (MIF), macrophage inhibitory protein (MIP)–1α, MIP-1β, nerve growth factor-beta (NGF-β), platelet derived growth factor (PDGF-ββ), regulated upon activation normal T cell expressed and presumably secreted (RANTES),stem cell factor (SCF), stem cell growth factor-beta (SCGF-β), stromal cell-derived factors 1- alpha (SDF-1α), tumor necrosis factor alpha (TNF)–α, TNF-β, TNF-related apoptosis-inducing ligand (TRAIL), and vascular endothelial growth factor (VEGF). Supernatants were filtered and centrifuged prior to testing. Standard curves were used to calculate cytokine concentrations in the samples using a 5-parameter logistic regression model. The inter-and intra-assay variability was performed by comparing cytokine concentrations in replicates plated on the same or across plates, with Spearman r values >0.8 considered acceptable per cytokine. Cytokine levels below the lower limit of detection of the assay were reported as the mid-point between zero and the lowest concentrations measured for each cytokine.

### Statistical Analysis

Descriptive data of continuous variables were summarized using medians and interquartile ranges, whilst categorical data were summarized with both frequency counts and percentages. A generalized estimating equation model using a binomial distribution and accounting for repeated measures was used to determine the effect of time on each binary clinical characteristic. Wilcoxon matched-pairs signed-ranks test was used to compare continuous data in two-time points. Linear mixed models were used to evaluate the associations between CSTs and inflammatory markers at women level with visits nested within a woman. The fixed term effects are CST, age, PSA, any STI and a constant term. The random effect are participant ID and time/visit. Time/visit was considered a categorical variable with three levels and an independent/simple covariance structure was assumed. In addition, linear mixed models were also used to estimate the effect of CSTs, metronidazole treatment, BV recurrence or persistence on the concentrations of each cytokine, adjusting for age, STI and recent sexual activity, as measured by PSA. Principal component analysis (PCA) was used to obtain summary measures for the multivariable cytokine set and performed using the FactoMineR package (https://cran.r-project.org/web/packages/FactoMineR/index.html) in R (www.r-project.org). Relative abundance of the most common taxa in cervicovaginal bacterial communities and log_10_-transformed fold change in various cytokines at baseline and after treatment were used for PCA, as previous described (14). The cos2 value of the variable indicates its contribution in driving the input data into principal components. Pearson correlation was used to determine associations between factors and principal components. All statistical calculations were carried out using R and SAS version 9.4 (SAS Institute Inc., Cary, NC, USA). Adjustment of multiple comparisons was performed using False Discovery Rate (FDR) (31). P-values less than 0.05 were considered statistically significant.

## Results

### Characteristics of Study Participants

Of the 101 HIV-negative women enrolled in the CAPRISA 083 cohort, 56 women with median age of 24 years [interquartile range (IQR) 21-27 years] had symptomatic intermediate BV or BV by Nugent score (NS) (Nugent-BV defined as NS ≥4) at baseline, who attended all three visits, and had genital specimen stored to conduct the longitudinal microbiota and cytokine analysis were used for this sub-analysis (**Figure 1**). At baseline, all 56 women received treatment with a single dose of 2g metronidazole. Twenty-eight of the 56 women also had sexually transmitted infections (STIs) and were treated with 1g of azithromycin (n=17), 2g of metronidazole (n=5), and 250mg (n=6) of ceftriaxone. Characteristics of study participants at all time points of sampling are reported in **Table 1**.

**Table 1:** Clinical characteristics of study population at baseline and follow-up (N=56).

Variable	Level	Baseline	Week 6	Week 12	P-value
		% (n/N)			
Age (years)	Median (IQR)	24 (21-27)	–	–	–
Condom use	Yes	71.4 (40)	–	–	–
	No	28.6 (16)	–	–	–
Frequency of condom use	Always	5.3 (3)	–	–	–
	Sometimes	66.1 (37)	–	–	–
	Never	28.6 (16)	–	–	–
Contraceptive use	Yes	37.5 (21)	–	–	–
	No	62.5 (35)	–	–	–
Type of contraception	Injection	52.4 (11/21)	–	–	–
	IUD	4.8 (1/21)	–	–	–
	Oral	14.3 (3/21)	–	–	–
	Subdermal implant	28.6 (6/21)	–	–	–
Genital examination	Abnormal	62.5 (35)	–	–	–
	Normal	37.5 (21)	–	–	–
Candidiasis	Yes	14.3 (8)	12.5 (7)	19.6 (11)	0.451
	No	85.7 (48)	87.5 (49)	80.4 (45)	
BV status (Nugent score)	No BV (0-3)	0 (0)	30.4 (17)	23.2 (13)	<0.0001
	Intermediate BV (4-6)	39.3 (22)	33.9 (19)	46.4 (26)	
	BV (7-10)	60.7 (34)	35.7 (20)	30.4 (17)	
*Chlamydia trachomatis*	Yes	30.4 (17)	5.4 (3)	3.4 (2)	0.001
	No	69.6 (39)	94.6 (53)	96.4 (54)	
*Neisseria gonorrhea*	Yes	10.7 (6)	0.0 (0)	1.8 (1)	0.113
	No	89.3 (50)	100 (56)	98.2 (55)	
*Trichomonas vaginalis*	Yes	8.9 (5)	3.6 (2)	0.0 (0)	0.013
	No	91.1 (51)	96.4 (54)	100 (56)	
Any STI	Yes	50.0 (28)	8.9 (5.0)	5.4 (3)	<0.0001
	No	50.0 (28)	91.1 (51)	94.6 (53)	
Co-conditions	BV only	51.8 (29)	60.7 (34)	73.2 (41)	–
	STI only	0.0 (0)	0.0 (0)	1.8 (1)	
	BV and STI	48.2 (27)	8.9 (5)	3.6 (2)	
	No BV or STI^-^	0 (0)	30.4 (17)	21.4 (12)	
PSA	Yes	19.6 (11)	21.4 (12)	25 (14)	0.785
	No	80.3 (45)	78.6 (44)	75 (42)	

BV, bacterial vaginosis; IQR, interquartile range; IUD, intrauterine device; PSA, prostate specific antigen; STI, sexually transmitted infection. Any STI includes all STIs tested excluding candidiasis.

### Changes in the Vaginal Community State Types Following Metronidazole Treatment

**Figure 2:**
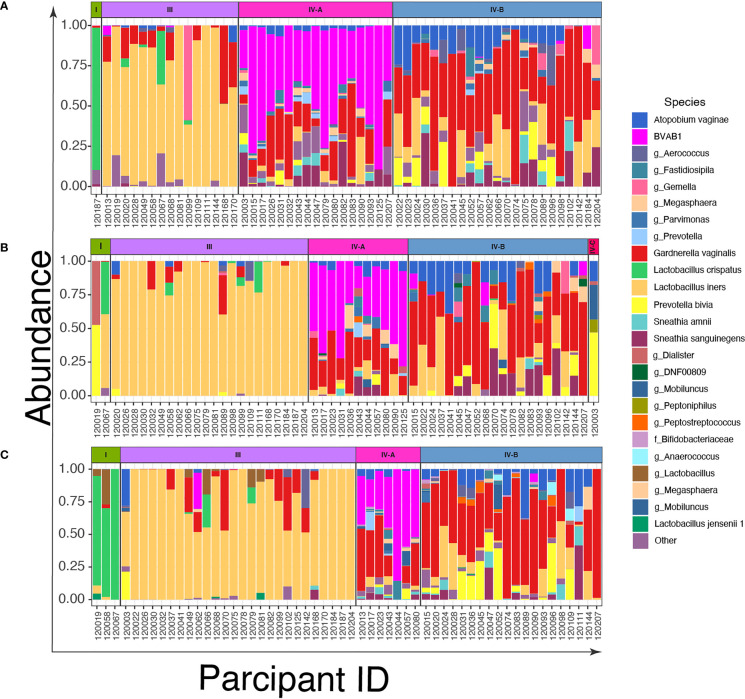
Bar plots of the relative abundance of the twenty most common taxa in cervicovaginal bacterial communities in women (n=56) before and after treatment with metronidazole with and without sexually transmitted infection (STI) treatment **(A)** at baseline; **(B)** at 6 weeks post-treatment, and **(C)** at 12 weeks post-treatment.

To describe the vaginal CSTs, we sequenced the V3-V4 region of the 16S rRNA gene of bacterial DNA in swabs collected from the women. We identified three CSTs based on the composition and relative abundance of bacterial species by VALENCIA (**Figure 2** and **Supplementary Figure 1**). These were CST I dominated by *L. crispatus*, CST III dominated by *L. iners*, and CST IV characterized by a wide array of strict and facultative anaerobes without a consistent dominant species. These CSTs could be further divided into sub-CSTs: CST IV was sub-divided into CST IV-A representing samples with high relative abundance of BVAB1, CST IV-B characterized by *G. vaginalis* dominance and CST IV-C characterized by an even community with moderate abundance of *Prevotella bivia*.

Among the 56 women, only 1/56 (2%) were assigned to CST I and 15/56 (27%) to CST III (**Figure 2A**), while the most prevalent CSTs at baseline were CST IV-A (30%, 17/56) and IV-B (41%, 23/56). Six weeks after treatment, the number of women with *L. iners (*CST III) increased to 39% (22/56) from 27% (**Figure 2B**), CST IV-A decreased to 20% (11/56) from 30% and CST IV-B remained relatively stable (41% (23/56) at baseline *versus* 36% (20/56) at 6-weeks). At 12 weeks post-treatment, the proportion of women with CST I vaginal microbiota increased slightly to 5% (3/56) and CST III (46%, 26/56) was the most common CST. CST IV-A reduced further to 12.5% (7/56), while the proportion of CST IV-B again remained relatively stable (36%, 20/56) (**Figure 2C**).

### Changes in the Vaginal Community State Types Following Antibiotic Treatment for STIs

We also determined whether the STI treatment had any effect on the vaginal microbiota by assessing CSTs of six women treated with azithromycin for present or suspected infection with *Chlamydia trachomatis*. All six women did not have BV by Nugent score at baseline and were excluded from the downstream analysis. **Supplementary Figure 2** depicts the fifteen most common bacterial species shown per patient. At baseline, 2/6 women had *L. iners* dominated vaginal microbiota, another 2/6 had communities dominated by both BVAB1 and *G. vaginalis* while the other 2 had a by a wide array of bacterial without a consistent dominant species. At 6 weeks after treatment, only 1 woman remained dominated by *L. iners* with while 2 changed to an *L. crispatus* dominated microbiota. *L. iners* became a dominant species in three women 12 weeks after treatment. One woman did not have 16S data at 6 weeks and 3 did not have at 12 weeks after treatment.

### Intra-Individual Transitions Across CSTs

**Figure 3:**
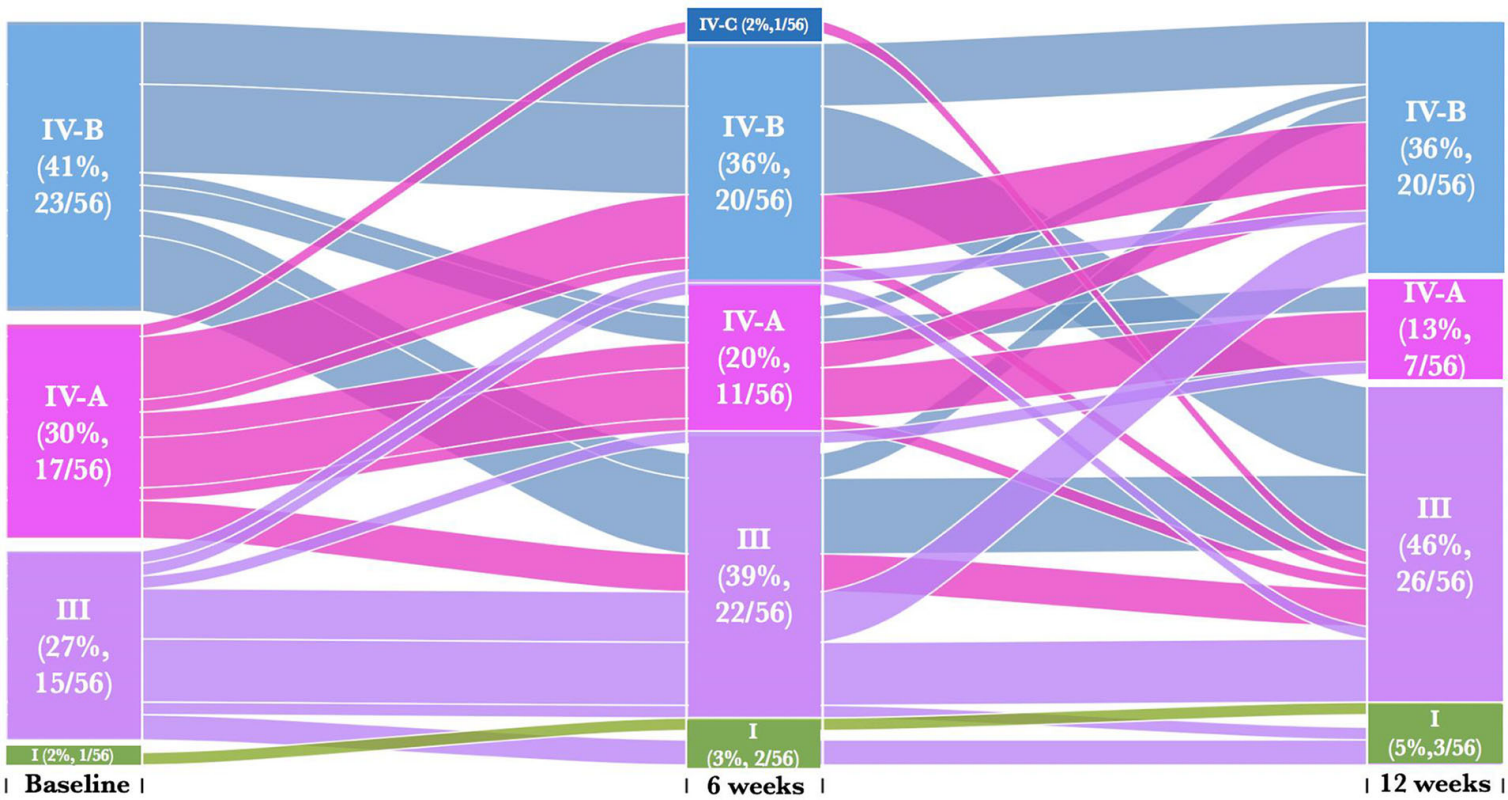
Alluvial diagrams are showing participant transition (n=56) between CSTs pre- and post-treatment. CST I (green) lacked a consistent dominant species, but all communities included *L. crispatus*, CST III (purple) *L. iners* dominance, CST IV-A (pink) had diverse bacterial communities dominated by BVAB1, CST IV-B characterized by *G. vaginalis* dominance and CST IV-C characterized by an even community with moderate abundance of *Prevotella bivia*.

We next assessed whether metronidazole treatment caused individual participant transitions between CSTs, particularly towards *Lactobacillus* dominated CSTs. We found that 25% (14/56) of women remained in their baseline CST [CST III (36%, 5/14), CST IV-A (29%, 4/14), CST IV-B (36%, 5/14)] despite treatment (**Figure 3** and **Supplementary Table 1A**). Twenty percent (11/56) of women transitioned from a profile of BV-associated CSTs [CST IV-A (27%, 3/11) or CST IV-B (73%, 8/11)] at baseline into CST III at 6 weeks post-treatment and only 18% (2/11) reverted back CST IV-B and none for CST IV-A. Furthermore, 27% (15/56) transitioned across CSTs at 12 weeks post-treatment despite remaining in their baseline CSTs at the 6-week follow-up visit. These include 27% (4/15) women that transition from CST III to CST IV-B, 7% (1/15) from CST III to CST I, 7% (1/15) from CST IV-A to CST III, 13% (2/15) from CST IV-A to CST IV-B and 47% (7/15) from CST IV-B to CST III. The highest percentage of women that transition to CST III at 12 weeks had CST IV-B (9/20) at the baseline. None of these individual participant transitions between CSTs were significant using pairwise symmetry tests (**Supplementary Table 1B**). Furthermore, women who transitioned from one CST at baseline to another at 6 weeks post treatment had BV and/or STIs, with no distinct separations between those who transitioned compared to those who did not transitioned.

We then assessed whether the women who transitioned between CSTs from baseline to 6 weeks post-treatment either (i) remained in their new CSTs, (ii) reverted to their baseline CSTs, or (iii) transitioned to other CST at 12 weeks post-treatment (**Figure 3**). Only 36% (20/56) of women remained in their new CSTs following treatment, and did not transition to another CST [CST I (10%, 2/20), CST III (45%, 9/20), CST IV-A (15%, 3/20), CST IV-B (25%, 5/20)] at 12 weeks post-treatment. Seven percent (4/56) of women initially changed their CST at baseline to another CST at 6 weeks, and then transitioned back to baseline CST [CST III (25%, 1/4), CST IV-B (75%, 3/4)] at 12 weeks. Only 5% (3/56) of women who transitioned between CSTs from baseline to 6 weeks post-treatment further transitioned to other CST [CST III (67%, 2/3), CST IV-B (33%, 1/3)] at 12 weeks post-treatment. There were no significant differences amongst women who transitioned between baseline and follow up visits CSTs (**Supplementary Table 1**).

### Relationship Between CSTs and Genital Tract Cytokines

To test the hypothesis that vaginal microbiota dominated with non-*Lactobacillus* species would be associated with increased cytokine concentrations compared to communities dominated by *Lactobacillus* species, we performed linear mixed effect regression analysis comparing genital cytokine concentrations with prevalent CSTs over time. Here, CST I and CST III were combined to represent the *Lactobacillus* group and to avoid analysing using CST I number alone. Women with vaginal CST IV-A communities had higher concentrations of hepatocyte growth factor interleukin (IL-5, β= 0,651, 95% confidence interval (CI) 0,079 – 1,223; p= 0,026), IL-7 (β= 0,518, 95% CI 0,108 – 0,928; p= 0,013), IL-18 (β= 0,643, 95% CI 0,181– 1,105; p= 0,006), and leukemia inhibitory factor (LIF, β= 0,620, 95% CI 0,227– 1,012; p= 0,002); compared to those with *Lactobacillus* dominance (CST I and CST III) and lower concentrations of growth-regulated oncogene-alpha (GRO-α, β= -0,722, 95% CI -1,262– (-0,182); p= 0,009), IL-12p40 (β= -1,288, 95%CI -1,989– (-0,588); p= <0.0001), interferon-γ -inducible protein (IP)-10 (β= -1,378, 95%CI -2,048 – (-0,709); p= <0.0001), monokine induced by gamma- interferon (MIG, β= -0,631, 95% CI -1,036 – (-0,227); p= 0.002), RANTES (β= -0,682, 95% CI -1,323 – (-0,041); p= 0.037) and stem cell factor (SCF, β= -1,099, 95% CI -1,906 – (-0,291); p= 0,008) (**Supplementary Figure 2** and **Supplementary Table 2**), adjusting for age, recent sexual activity and STIs. Women with vaginal CST IV-B communities had higher concentrations of IL-1α (β= 0.890, 95% CI 0.011 – 1.786; p= 0.044), IL-18 (β= 0,589, 95% CI 0,220 – 0,957; p=0,002), LIF (β= 0,466, 95% CI 0,149– 0,782; p= 0.004) and macrophage migration inhibitory factor (MIF, β= 0,610, 95% CI 0,261 – 0,960; p= 0,001) compared to women with *Lactobacillus* dominance (CST I and CST III) and reduced concentrations of GROa (β= -0,670, 95% CI -1,100 – (-0,241); p= 0,002), IP-10 (β= -1,369, 95% CI -1,881– (-0,857); p= <0.0001) and MIG (β= -0,381, 95% CI -0,690 – (-0,072); p= 0,016), respectively (**Supplementary Figure 2**).

### Impact of Metronidazole Treatment on Temporal Dynamics of Mucosal Cytokines Profiles

We next assessed whether women who cleared BV (NS <4) at week 6 post-treatment and did not recur by week 12 also had lower genital cytokines. Compared to pre-treatment samples, a significant reduction in concentrations of certain cytokines was noted in post-treatment SoftCup supernatants of women who cleared BV, including tumor necrosis factor (TNF)-α (p= 0.027), IL-1β (p= 0.002); IL-8 (p= 0.040); LIF (p= 0.043). In contrast, cytokine granulocyte macrophage colony-stimulating factor (GM–CSF, p= 0.033) increased following metronidazole treatment (**Supplementary Figure 3**).

**Figure 4:**
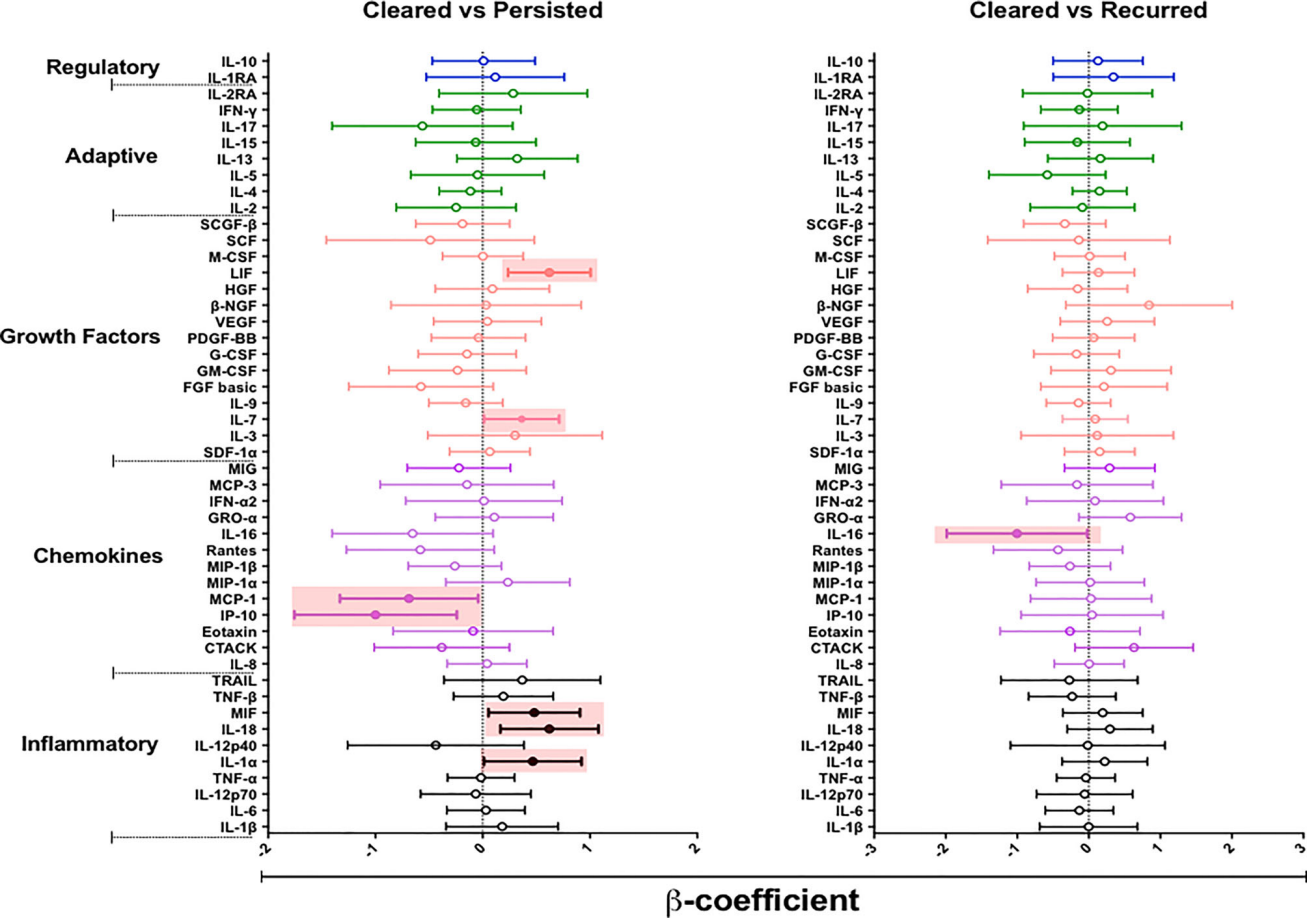
β-coefficients from linear mixed models to determine the effect of persistent (n= 35) or recurrent BV (n= 9) on the cytokine milieu compared to women who cleared (n= 13) their BV. Individual associations are shown between BV status and pro-inflammatory cytokines (black), chemokines (purple), growth factors (orange), adaptive response cytokines (green), and regulatory cytokines (blue), with error bars depicting standard error. Shaded shapes represent significant associations, after adjusting for age, recent sexual activity, and sexually transmitted infections.

We hypothesized that episodes of BV persistence or recurrence may distinctly alter mucosal cytokine and chemokine concentrations compared to BV clearance. To test our hypothesis, we used a linear mixed model to compare the concentrations of cytokines in women who cleared BV (at 12 weeks post-treatment) with those who had persistent BV (at 12 weeks post-treatment) (**Figure 4** and **Supplementary Table 3**). After adjusting for age, recent sexual activity and STIs, we observed higher concentrations of several cytokines in women with persistent BV relative to the BV cleared group: including IL-1α (*β*= 0.466, 95% CI 0.011 – 0.921; p= 0.045), IL-18 (β= 0.621, 95% CI 0.164 – 1.078; p= 0.009), MIF (β= 0.481, 95% CI 0.054 – 0.908; p= 0.028), IL-7 (β= 0.363, 95% CI 0.012 – 0.713; p= 0.043), and LIF (β= 0.621, 95% CI 0.236 – 1.005; p= 0.038). Interestingly, women who tended to experience persistent BV also had significantly lower concentrations of IP-10 (β= -0.999, 95% CI -1.757 – (-0.241); p= 0.011) and monocyte chemotactic protein (MCP)-1 (β= -0.687, 95% CI -1.331 – (-0.043); p= 0.037) relative to those who cleared BV. However, none of these associations remained significant after adjusting for multiple comparisons.

Next, we assessed whether BV recurrence at 12 weeks post-treatment, after initial BV clearance at 6 weeks, influenced changes in mucosal cytokines relative to BV clearance by week 12. Only IL-16 (β= -1.004, 95% CI -1.986 – (-0.022); p= 0.045) appeared to be significantly reduced in women who had BV recurrence relative to those who cleared BV, although not after adjustment for confounders.

## Relationship Between Vaginal Microbial Taxa and Cytokines

**Figure 5:**
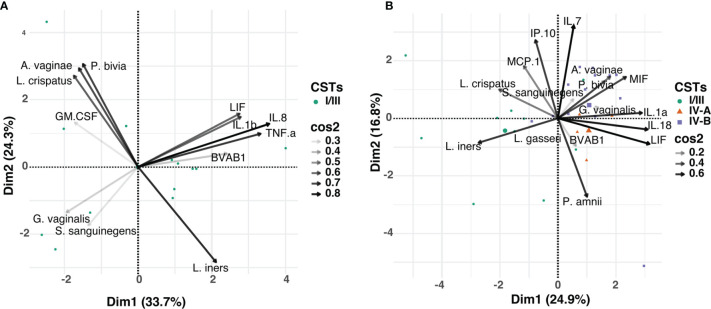
Principal component analysis (PCA) showing the relationship between the relative abundance of the individual bacterial taxa and mucosal cytokines associated with BV, in: **(A)** women that cleared BV at 12 weeks post-treatment (n=13), and **(B)** those that had persistent BV (n=35). Data points represent the projection of participants on PCs 1 and 2 with colour and shape corresponding to their respective CST assignment. The arrows correspond to eigenvectors, which give a sense of the magnitude of the factors in the data set that drive the separation. The arrow length indicates the variance across the dataset and the angle between the arrows describes the correlation between the variables. The cos2 value of the variable indicates its contribution in driving the input data into principal components. Variables with large cos2 values contribute more to the distance separating the data points in the PCA.

Focusing on the most abundant vaginal bacterial taxa across all three visits and the cytokines that were significantly associated with BV clearance (including TNF-α, IL-1β, IL-8, LIF, GM-CSF), we determined whether the observed alterations in cytokine concentrations post-treatment were associated with relative abundance of any specific bacterial taxa (**Figure 5A**). At any time point, concentrations of TNF-α, IL-1β, IL-8, and LIF appeared to be influenced by the relative abundance of BVAB1, commonly found in women with BV. In contrast, GM-CSF appeared to be associated with the abundance of *L. crispatus*. Next, we used cytokines (IL-1α, IL-7, IL-18, IP-10, MCP-1, MIF and LIF) that were significantly associated with persistent BV and correlated with the relative abundance of the 20 most abundant vaginal bacterial taxa found in both CST IV-A and CST IV-B. At any time point, concentrations of IL-1α, IL-18, MIF, and LIF clustered with the relative abundance of *G. vaginalis*, also commonly found in women with BV (**Figure 5B**). Furthermore, the relative abundance of *L. crispatus* was clustered with MCP-1 and IP-10 in women with persistent BV.

## Discussion

BV is common among women of African descent (32) and is often recalcitrant to treatment with metronidazole or clindamycin (33). However, little is known about the temporal relationships between microbial shifts and genital inflammation. Here, we investigated the effects of metronidazole treatment on the vaginal microbiota and concentrations of mucosal soluble immune mediators in women pre- and post-treatment for BV, including those who had treatment failure or BV recurrence. We found that complete BV clearance rates were suboptimal and recurrence rates were high post-treatment. The post-treatment *16S rRNA gene sequencing* data showed that treatment mediated a gradual shift away from a BV-associated strict anaerobic species dominated profile towards an increased relative abundance of *Lactobacillus* species, particularly *L. iners*. Women classified as having persistent BV had high concentrations of mucosal cytokines and chemokines relative to BV clearance and this response was likely driven by BV-associated strict anaerobes.

In agreement with previous studies that used Nugent scoring to diagnose BV (34) clinical, we observed short-term BV clearance (NS <4) rates in our cohort, with more than half of those who cleared BV by 6 weeks experiencing recurrence (NS ≥4) by 12 weeks post-treatment, suggesting that stability in the vaginal microbiota was not achieved after short-term gains. Furthermore, the majority of women experienced persistent BV despite treatment. Metronidazole treatment resulted in the decreased prevalence of diverse CSTs and a shift toward the increased proportion of women having *L. iners* dominant CST III. The increase in the relative abundance of *L. iners* is consistent with earlier studies showing a positive correlation between the abundance of lactobacilli species and metronidazole treatment (11, 13). While metronidazole treatment was also associated with a shift toward increased relative abundance of *L. iners*, the relative abundance of *L. crispatus* was extremely modest. The metronidazole induced *L. iners*-dominant state, an unstable community, with high probability of transitioning back to a high diversity state is particularly concerning and may explain high rates of BV occurrence and recurrence among women of sub-Saharan Africa descent. Our findings emphasize a need to extensively investigate *L. iners* role in both lactobacilli-dominated and diverse microbial communities. Given the specific health benefits afforded by a Lactobacillus-dominant vaginal microbiota against infections and the limited efficacy of antibiotics against BV-associated sequelae, a viable alternative such as probiotics to improve Lactobacillus representation as part of curative or preventive vaginal dysbiosis interventions are needed. In addition, there is a need to include rapid molecular assays that are able to quantify key beneficial bacteria and those with pathogenic potential, including biofilm producers in a point of care management.

In this cohort of African women with BV, BVAB1, and *G. vaginalis* were found to be the most abundant species in the non-*Lactobacillus*-dominated CSTs, with BVAB1 being the most easily cleared by metronidazole treatment (35). Metronidazole treatment was associated with the reduced relative abundance of *G. vaginalis*, but the extent of the reduction was modest and appeared to re-establish at 12 weeks post-treatment. This was confirmed by the number of women who transitioned out of CST IVA or B into CST III, but reverted back to either of their original CSTs at 12 weeks post-treatment. Our findings are consistent with other studies that suggested that women with a high *G. vaginalis* (36) were the most likely to fail treatment. Studies have hypothesized that metronidazole may be ineffective against *G. vaginalis* due to their ability to form complex biofilms and/or harbor metronidazole resistance (37, 38). Another study suggests that high levels of CRISPR-associated Cas genes can undermine the efficacy of metronidazole against *G. vaginalis* (39). Although more longitudinal studies are needed to understand the effect of treatment on BV, these findings suggest that treatment may successfully eradicate BV in women colonized by BVAB1 communities.

Recent studies showed the effect of azithromycin on the vaginal microbiome. Although limited by sample size, our findings are in agreement with other studies that showed a shift towards increased relative abundance of lactobacilli (mainly *L. iners*) following 1g of oral azithromycin treatment. It has been shown that women with high relative abundance of *L. iners* were associated with increased susceptibility to *C. trachomatis* infection, suggesting that azithromycin treatment increase probabilities of transitioning to diverse disease-associated states or reinfection (40, 41). Considering that both metronidazole and azithromycin favor increased relative abundance of unstable *L. iners* communities, there is a need to use Gardnerella specific-biofilm dissolving treatment, boosted with Lactobacilli-based live biotherapeutic products to restore an optimal vaginal microbiota after antibiotic treatment.

We determined whether vaginal microbiota dominated with non-*Lactobacillus* species is associated with increased genital cytokine production using linear mixed effect regression analysis. In agreement with previous studies (42, 43), we demonstrated that high concentrations of proinflammatory cytokines, adaptive growth factors and hematopoietic cytokines (IL-1α, IL-5, IL-7, IL-18, LIF and MIF) were associated with non-optimal vaginal microbiota while communities dominated by *Lactobacillus* species (mainly *L. iners* in this cohort) were associated with lower concentrations of inflammatory cytokines and chemokines (GRO-α, IL-12p40, IP-10, MIG, RANTES and SCF). In contrast, other studies reported that women with communities dominated by *L. iners* had increased concentrations of IP-10 (14, 43, 44). Pro-inflammatory cytokines and chemokines such as IL-12p40, IP-10, RANTES and are each involved in recruitment of T cells, neutrophil and antigen presenting cells to infection sites (45–48). Our results suggest that vaginal microbiota dominated by non-*iners*-*Lactobacillus* species, metronidazole induced communities dominated by *L. iners* may have similar beneficial effect on inflammatory response and HIV susceptibility.

Unlike previous studies that only investigated the impact of BV clearance with metronidazole at one-month post-treatment (14, 39), we also extended our analysis to determine the long-term impact of treatment on the vaginal microbiota and the impact of BV clearance, persistence and recurrence on mucosal cytokine concentrations. In this study, BV clearance was associated with decreased concentrations of TNF-α, IL-1β, IL-8, and LIF compared to matched pre-treatment visits. Our data suggest that treatment over time or BV clearance had a desirable effect by downregulating cytokines that induce an inflammatory response (LIF) and those that are linked to increased risk of acquiring HIV infection (TNF-α, IL-1β; IL-8) (4). In contrast, clearing BV was also associated with increased concentrations of the growth factor GM-CSF, which is known to induce proliferation and differentiation of hematopoietic cells such as neutrophils and macrophages in response to invading pathogen (49). Relative to cleared BV, persistent BV had reduced concentrations of cytokines that may influence the trafficking of lymphocytes (MCP-1 and IP-10). Our findings are consistent with previous studies that found *in vivo* IP-10 suppression concentrations in BV positive women (50–52). Studies suggest that IP-10 concentration are increased in viral infections, and decreased by pathogenic bacterial infection (51, 53, 54) but the role and significance of IP-10 in BV remains unclear. Persistence was also associated with increased concentrations of cytokines (IL-1α, IL-18, MIF, IL-7 and LIF) associated with HIV susceptibility. IL-1α, IL-18, and LIF are known to induce an inflammatory response and acute immune response, including the proliferation of Th17 type CD4+ cells (55). These findings suggest that BV persistence may alter lymphocyte chemotaxis and potentially increase susceptibility to HIV infection.

Although BV is a multifactorial condition, with no one organism being linked to increased or decreased concentrations of mucosal cytokines, our study showed that TNF-α, IL-1β, IL-18, MIF, and IL-8 appeared to be differentially regulated bychanges in the relative abundance of BVAB1 and *G. vaginalis*. This is consistent with previous studies from our group and others that found a correlation between Nugent-defined BV status (including high microbial diversity by 16S rRNA sequencing) in women and changes in pro-inflammatory cytokine and chemokine concentrations (4, 5, 44, 56). In contrast, the relative abundance of *L. crispatus* clustered with the concentrations of GM-CSF, MCP-1, and IP-10 confirming an anti-inflammatory effect mediated by lactobacilli species dominance (57, 58). Although these results suggest a relationship between vaginal microbiota and altered cytokine concentrations post-treatment, mechanistic studies are required to confirm this relationship further.

The strength of this study includes longitudinal assessment of the impact that efforts to normalize the vaginal microbiome have on mucosal cytokine concentrations in a prospective cohort of women undergoing STI and BV management. However, this study had limitations, including the sample size that restricted the ability to investigate in detail the effects of BV clearance, persistence, or recurrence on vaginal microbiota and how changes triggered by treatment would affect genital inflammation. Another limitation is that almost half of participants had STI and also treated with azithromycin and ceftriaxone and this may have confounded the results. The lack of the control group also limited our ability to investigate microbial and cytokine changes between the treatment experienced and naïve groups. We also did not investigate the role cellular markers of genital inflammation on BV clearance and persistence. Likewise, we were unable to investigate the impact of other potential co-factors, including human papillomavirus, herpes simplex virus, hormonal contraceptives, diets, hormonal status, other vaginal disorders (e.g., aerobic vaginitis) on microbiota, although these may have different biological effects.

In conclusion, metronidazole treatment induced a shortlived reduction of BV-associated microbiota and an increase in vaginal *Lactobacillus* species, which resulted in altered mucosal cytokine concentrations. Our findings suggest that the efficacy of BV treatment may depend on the pre-treatment microbial species present in the female genital tract, suggesting value in screening for biofilm-producing bacteria before administering treatment. Only when novel and more effective BV treatment regimes that target and disrupt biofilm-producing bacteria are developed will we be able to reduce the burden of BV.

## Data Availability Statement

The datasets presented in this study can be found in online repositories. The names of the repository/repositories and accession number(s) can be found in the article/**Supplementary Material**.

## Ethics Statement

The studies involving human participants were reviewed and approved by Ethics Review Committee of the University of KwaZulu-Natal (BREC number: BE403/16). The patients/participants provided their written informed consent to participate in this study.

## Author Contributions

AMt, SN, and LL conceived and designed the analysis and AMt, SN, SEJ, KM, JR and FO performed the analyses. AR, AMi and NG were the principal investigators of the CAPRISA 083 study. AMt, SN, JS, FO, NG, CB, JG, HO, KM, GM, TO, AR, AMi, SA, JR, J-AP, QA, HJ, and LL contributed to the interpretation and discussion of the results and writing of the manuscript. All authors contributed to the article and approved the submitted version.

## Funding

AMt received support from the CAPRISA Research Administration and Management Training Program (Grant # G11 TW010555-01). LL is funded by a SANTHE Path to Independence award, and a FLAIR Fellowship supported by the African Academy of Sciences and the Royal Society. SN was supported by Columbia University-Southern African Fogarty AITRP Programme (grant# D43TW00231), National Research Fund Thuthuka Research Grant (grant# TTK160510164586), and Poliomyelitis Research Foundation Research Grant (grant# 16/17).

## Conflict of Interest

The authors declare that the research was conducted in the absence of any commercial or financial relationships that could be construed as a potential conflict of interest.

## Publisher’s Note

All claims expressed in this article are solely those of the authors and do not necessarily represent those of their affiliated organizations, or those of the publisher, the editors and the reviewers. Any product that may be evaluated in this article, or claim that may be made by its manufacturer, is not guaranteed or endorsed by the publisher.

## References

[1] Buve A Jespers V Crucitti T Fichorova RN. The Vaginal Microbiota and Susceptibility to HIV. AIDS (2014) 28(16):2333–44. doi: 10.1097/QAD.0000000000000432 25389548

[2] Hayes R Watson-Jones D Celum C van de Wijgert J Wasserheit J. Treatment of Sexually Transmitted Infections for HIV Prevention: End of the Road or New Beginning? AIDS (2010) 24(Suppl 4):S15–26. doi: 10.1097/01.aids.0000390704.35642.47 PMC3827743 21042049

[3] Fettweis JM Serrano MG Brooks JP Edwards DJ Girerd PH Parikh HI. The Vaginal Microbiome and Preterm Birth. Nat Med (2019) 25(6):1012–21. doi: 10.1038/s41591-019-0450-2 PMC6750801 31142849

[4] Anahtar MN Byrne EH Doherty KE Bowman BA Yamamoto HS Soumillon M. Cervicovaginal Bacteria Are a Major Modulator of Host Inflammatory Responses in the Female Genital Tract. Immunity (2015) 42(5):965–76. doi: 10.1016/j.immuni.2015.04.019 PMC4461369 25992865

[5] Gosmann C Anahtar MN Handley SA Farcasanu M Abu-Ali G Bowman BA. Lactobacillus-Deficient Cervicovaginal Bacterial Communities Are Associated With Increased HIV Acquisition in Young South African Women. Immunity (2017) 46(1):29–37. doi: 10.1016/j.immuni.2016.12.013 28087240 PMC5270628

[6] Lennard K Dabee S Barnabas SL Havyarimana E Blakney A Jaumdally SZ. Microbial Composition Predicts Genital Tract Inflammation and Persistent Bacterial Vaginosis in South African Adolescent Females. Infect Immun (2018) 86(1)e00410–17. doi: 10.1128/IAI.00410-17 PMC5736802 29038128

[7] Shipitsyna E Roos A Datcu R Hallen A Fredlund H Jensen JS. Composition of the Vaginal Microbiota in Women of Reproductive Age–Sensitive and Specific Molecular Diagnosis of Bacterial Vaginosis Is Possible? PloS One (2013) 8(4):e60670. doi: 10.1371/journal.pone.0060670 23585843 PMC3621988

[8] Christiaens I Zaragoza DB Guilbert L Robertson SA Mitchell BF Olson DM. Inflammatory Processes in Preterm and Term Parturition. J Reprod Immunol (2008) 79(1):50–7. doi: 10.1016/j.jri.2008.04.002 18550178

[9] McCullough LE Miller EE Calderwood LE Shivappa N Steck SE Forman MR. Maternal Inflammatory Diet and Adverse Pregnancy Outcomes: Circulating Cytokines and Genomic Imprinting as Potential Regulators? Epigenetics (2017) 12(8):688–97. doi: 10.1080/15592294.2017.1347241 PMC5687326 28678596

[10] Doerflinger SY Throop AL Herbst-Kralovetz MM. Bacteria in the Vaginal Microbiome Alter the Innate Immune Response and Barrier Properties of the Human Vaginal Epithelia in a Species-Specific Manner. J Infect Dis (2014) 209(12):1989–99. doi: 10.1093/infdis/jiu004 24403560

[11] Bradshaw CS Morton AN Hocking J Garland SM Morris MB Moss LM. High Recurrence Rates of Bacterial Vaginosis Over the Course of 12 Months After Oral Metronidazole Therapy and Factors Associated With Recurrence. J Infect Dis (2006) 193(11):1478–86. doi: 10.1086/503780 16652274

[12] Barrons R Tassone D. Use of Lactobacillus Probiotics for Bacterial Genitourinary Infections in Women: A Review. Clin Ther (2008) 30(3):453–68. doi: 10.1016/j.clinthera.2008.03.013 18405785

[13] Verwijs MC Agaba SK Darby AC van de Wijgert J. Impact of Oral Metronidazole Treatment on the Vaginal Microbiota and Correlates of Treatment Failure. Am J Obstet Gynecol (2019) 222(2):157.e1–157.e13. doi: 10.1016/j.ajog.2019.08.008 31404542 PMC6995998

[14] Joag V Obila O Gajer P Scott MC Dizzell S Humphrys M. Impact of Standard Bacterial Vaginosis Treatment on the Genital Microbiota, Immune Milieu, and *Ex Vivo* Human Immunodeficiency Virus Susceptibility. Clin Infect Dis (2019) 68(10):1675–83. doi: 10.1093/cid/ciy762 PMC6495022 30407498

[15] Mayer BT Srinivasan S Fiedler TL Marrazzo JM Fredricks DN Schiffer JT. Rapid and Profound Shifts in the Vaginal Microbiota Following Antibiotic Treatment for Bacterial Vaginosis. J Infect Dis (2015) 212(5):793–802. doi: 10.1093/infdis/jiv079 25676470 PMC4539900

[16] McClelland RS Richardson BA Hassan WM Chohan V Lavreys L Mandaliya K. Improvement of Vaginal Health for Kenyan Women at Risk for Acquisition of Human Immunodeficiency Virus Type 1: Results of a Randomized Trial. J Infect Dis (2008) 197(10):1361–8. doi: 10.1086/587490 PMC4122228 18444793

[17] Balkus JE Richardson BA Mandaliya K Kiarie J Jaoko W Ndinya-Achola JO. Establishing and Sustaining a Healthy Vaginal Environment: Analysis of Data From a Randomized Trial of Periodic Presumptive Treatment for Vaginal Infections. J Infect Dis (2011) 204(2):323–6. doi: 10.1093/infdis/jir241 PMC3114467 21673045

[18] Balkus JE Manhart LE Lee J Anzala O Kimani J Schwebke J. Periodic Presumptive Treatment for Vaginal Infections May Reduce the Incidence of Sexually Transmitted Bacterial Infections. J Infect Dis (2016) 213(12):1932–7. doi: 10.1093/infdis/jiw043 PMC4878720 26908758

[19] Cohen CR Wierzbicki MR French AL Morris S Newmann S Reno H. Randomized Trial of Lactin-V to Prevent Recurrence of Bacterial Vaginosis. New Engl J Med (2020) 382(20):1906–15. doi: 10.1056/NEJMoa1915254 PMC7362958 32402161

[20] Garrett NJ Osman F Maharaj B Naicker N Gibbs A Norman E. Beyond Syndromic Management: Opportunities for Diagnosis-Based Treatment of Sexually Transmitted Infections in Low- and Middle-Income Countries. PloS One (2018) 13(4):e0196209. doi: 10.1371/journal.pone.0196209 29689080 PMC5918163

[21] Choe HS Lee DS Lee SJ Hong SH Park DC Lee MK. Performance of Anyplex II Multiplex Real-Time PCR for the Diagnosis of Seven Sexually Transmitted Infections: Comparison With Currently Available Methods. Int J Infect Dis IJID Off Publ Int Soc Infect Dis (2013) 17(12):e1134–40. doi: 10.1016/j.ijid.2013.07.011 24095619

[22] Department of Health. Sexually Transmitted Infections Management Guidlines. Pretoria: South African Department of Health South Africa (2015). p. 1–28. Available from: https://www.idealclinic.org.za/docs/National-Priority-Health-Conditions/Sexually%20Transmitted%20Infections_%20Management%20Guidelines%202015.pdf (Accessed: 12 June 2020).

[23] Nugent RP Krohn MA Hillier SL. Reliability of Diagnosing Bacterial Vaginosis is Improved by a Standardized Method of Gram Stain Interpretation. J Clin Microbiol (1991) 29(2):297–301. doi: 10.1128/jcm.29.2.297-301.1991 1706728 PMC269757

[24] McKinnon LR Achilles SL Bradshaw CS Burgener A Crucitti T Fredricks DN. The Evolving Facets of Bacterial Vaginosis: Implications for HIV Transmission. AIDS Res Hum Retroviruses (2019) 35(3):219–28. doi: 10.1089/aid.2018.0304 PMC6434601 30638028

[25] Callahan BJ McMurdie PJ Rosen MJ Han AW Johnson AJ Holmes SP. DADA2: High-Resolution Sample Inference From Illumina Amplicon Data. Nat Methods (2016) 13(7):581–3. doi: 10.1038/nmeth.3869 PMC4927377 27214047

[26] McMurdie PJ Holmes S. Phyloseq: An R Package for Reproducible Interactive Analysis and Graphics of Microbiome Census Data. PloS One (2013) 8(4):e61217. doi: 10.1371/journal.pone.0061217 23630581 PMC3632530

[27] France MT Ma B Gajer P Brown S Humphrys MS Holm JB. VALENCIA: A Nearest Centroid Classification Method for Vaginal Microbial Communities Based on Composition. Microbiome (2020) 8(1):166. doi: 10.21203/rs.2.24139/v1 33228810 PMC7684964

[28] Mckinney W. Data Structures for Statistical Computing in Python. In Proceedings of the 9th Python in Science Conference 2010 Jun 28 (2010) 445:51–56. doi: 10.25080/Majora-92bf1922-00a

[29] Yue JC Clayton MK. A Similarity Measure Based on Species Proportions. Commun Stat - Theory Methods (2005) 34(11):2123–31. doi: 10.1080/STA-200066418

[30] Archary D Liebenberg LJ Werner L Tulsi S Majola N Naicker N. Randomized Cross-Sectional Study to Compare HIV-1 Specific Antibody and Cytokine Concentrations in Female Genital Secretions Obtained by Menstrual Cup and Cervicovaginal Lavage. PloS One (2015) 10(7):e0131906. doi: 10.1371/journal.pone.0131906 26147923 PMC4492781

[31] Benjamini Y Hochberg Y. On the Adaptive Control of the False Discovery Rate in Multiple Testing With Independent Statistics. J Educ Behav Stat (2000) 25(1):60–83. doi: 10.3102/10769986025001060

[32] van de Wijgert JH Borgdorff H Verhelst R Crucitti T Francis S Verstraelen H. The Vaginal Microbiota: What Have We Learned After a Decade of Molecular Characterization? PloS One (2014) 9(8):e105998. doi: 10.1371/journal.pone.0105998 25148517 PMC4141851

[33] Bukusi EA Cohen CR Meier AS Waiyaki PG Nguti R Njeri JN. Bacterial Vaginosis: Risk Factors Among Kenyan Women and Their Male Partners. Sex Transm Dis (2006) 33(6):361–7. doi: 10.1097/01.olq.0000200551.07573.df 16547451

[34] Lambert JA John S Sobel JD Akins RA. Longitudinal Analysis of Vaginal Microbiome Dynamics in Women With Recurrent Bacterial Vaginosis: Recognition of the Conversion Process. PloS One (2013) 8(12):e82599. doi: 10.1371/journal.pone.0082599 24376552 PMC3869700

[35] Srinivasan S Morgan MT Liu C Matsen FA Hoffman NG Fiedler TL. More Than Meets the Eye: Associations of Vaginal Bacteria With Gram Stain Morphotypes Using Molecular Phylogenetic Analysis. PloS One (2013) 8(10):e78633. doi: 10.1371/journal.pone.0078633 24302980 PMC3840219

[36] Macklaim JM Fernandes AD Di Bella JM Hammond JA Reid G Gloor GB. Comparative Meta-RNA-Seq of the Vaginal Microbiota and Differential Expression by Lactobacillus Iners in Health and Dysbiosis. Microbiome (2013) 1(1):12. doi: 10.1186/2049-2618-1-12 24450540 PMC3971606

[37] Hardy L Cerca N Jespers V Vaneechoutte M Crucitti T. Bacterial Biofilms in the Vagina. Res Microbiol (2017) 168(9-10):865–74. doi: 10.1016/j.resmic.2017.02.001 28232119

[38] Bradshaw CS Brotman RM. Making Inroads Into Improving Treatment of Bacterial Vaginosis - Striving for Long-Term Cure. BMC Infect Dis (2015) 15:292. doi: 10.1186/s12879-015-1027-4 26219949 PMC4518586

[39] Deng ZL Gottschick C Bhuju S Masur C Abels C Wagner-Dobler I. Metatranscriptome Analysis of the Vaginal Microbiota Reveals Potential Mechanisms for Protection Against Metronidazole in Bacterial Vaginosis. mSphere (2018) 3(3)e00262–18. doi: 10.1128/mSphereDirect.00262-18 PMC5990888 29875146

[40] Tamarelle J Ma B Gajer P. Nonoptimal Vaginal Microbiota After Azithromycin Treatment for Chlamydia trachomatis Infection. J Infect Dis. (2020) 221(4):627–35. doi: 10.1038/s41598-018-38253-4 PMC7530545 31573603

[41] Ahrens P Andersen LO Lilje B. Changes in the Vaginal Microbiota Following Antibiotic Treatment for Mycoplasma Genitalium, Chlamydia Trachomatis and Bacterial Vaginosis. PLoS One (2020) 15(7):e0236036. doi: 10.1038/s41598-018-38253-4 32722712 PMC7386580

[42] Masson L Arnold KB Little F Mlisana K Lewis DA Mkhize N. Inflammatory Cytokine Biomarkers to Identify Women With Asymptomatic Sexually Transmitted Infections and Bacterial Vaginosis Who Are at High Risk of HIV Infection. Sex Transm Infect (2016) 92(3):186–93. doi: 10.1136/sextrans-2015-052072 PMC6801014 26511781

[43] Shannon B Yi TJ Perusini S Gajer P Ma B Humphrys MS. Association of HPV Infection and Clearance With Cervicovaginal Immunology and the Vaginal Microbiota. Mucosal Immunol (2017) 10(5):1310–9. doi: 10.1038/mi.2016.129 PMC5526752 28120845

[44] Jespers V Kyongo J Joseph S Hardy L Cools P Crucitti T. A Longitudinal Analysis of the Vaginal Microbiota and Vaginal Immune Mediators in Women From Sub-Saharan Africa. Sci Rep (2017) 7(1):11974. doi: 10.1038/s41598-017-12198-6 28931859 PMC5607244

[45] Cooper AM Khader SA. IL-12p40: An Inherently Agonistic Cytokine. Trends Immunol (2007) 28(1):33–8. doi: 10.1016/j.it.2006.11.002 17126601

[46] Wira CR Fahey JV Sentman CL Pioli PA Shen L. Innate and Adaptive Immunity in Female Genital Tract: Cellular Responses and Interactions. Immunol Rev (2005) 206:306–35. doi: 10.1111/j.0105-2896.2005.00287.x 16048557

[47] Abbas AK Lichtman AH. Cellular and Molecular Immunology. 6th edn. Philadelphia: Saunders Elsevier Inc. (2007). p. 97–111.

[48] Persson-Dajotoy T Andersson P Bjartell A Calafat J Egesten A. Expression and Production of the CXC Chemokine Growth-Related Oncogene-α by Human Eosinophils. J Immunol (2003) 170(10):5309–16. doi: 10.4049/jimmunol.170.10.5309 12734381

[49] Gomez-Cambronero J Horn J Paul CC Baumann MA. Granulocyte-Macrophage Colony-Stimulating Factor Is a Chemoattractant Cytokine for Human Neutrophils: Involvement of the Ribosomal P70 S6 Kinase Signaling Pathway. J Immunol (2003) 171(12):6846–55. doi: 10.4049/jimmunol.171.12.6846 14662891

[50] Masson L Mlisana K Little F Werner L Mkhize NN Ronacher K. Defining Genital Tract Cytokine Signatures of Sexually Transmitted Infections and Bacterial Vaginosis in Women at High Risk of HIV Infection: A Cross-Sectional Study. Sex Transm Infect (2014) 90(8):580–7. doi: 10.1136/sextrans-2014-051601 25107710

[51] Hoermannsperger G Clavel T Hoffmann M Reiff C Kelly D Loh G. Post-Translational Inhibition of IP-10 Secretion in IEC by Probiotic Bacteria: Impact on Chronic Inflammation. PloS One (2009) 4(2):e4365. doi: 10.1371/annotation/583d95a8-c18c-4c66-92be-1b1505802d86 19197385 PMC2634842

[52] Kyongo JK Crucitti T Menten J Hardy L Cools P Michiels J. Cross-Sectional Analysis of Selected Genital Tract Immunological Markers and Molecular Vaginal Microbiota in Sub-Saharan African Women, With Relevance to HIV Risk and Prevention. Clin Vaccine Immunol CVI (2015) 22(5):526–38. doi: 10.1128/CVI.00762-14 PMC4412937 25761460

[53] Fichorova RN Buck OR Yamamoto HS Fashemi T Dawood HY Fashemi B. The Villain Team-Up or How Trichomonas Vaginalis and Bacterial Vaginosis Alter Innate Immunity in Concert. Sex Transm Infect (2013) 89(6):460–6. doi: 10.1136/sextrans-2013-051052 PMC3746192 23903808

[54] Jauregui CE Wang Q Wright CJ Takeuchi H Uriarte SM Lamont RJ. Suppression of T-Cell Chemokines by Porphyromonas Gingivalis. Infect Immun (2013) 81(7):2288–95. doi: 10.1128/IAI.00264-13 PMC3697598 23589576

[55] McKinnon LR Liebenberg LJ Yende-Zuma N Archary D Ngcapu S Sivro A. Genital Inflammation Undermines the Effectiveness of Tenofovir Gel in Preventing HIV Acquisition in Women. Nat Med (2018) 24:491–6. doi: 10.1038/nm.4506 PMC5893390 29480895

[56] Shannon B Gajer P Yi TJ Ma B Humphrys MS Thomas-Pavanel J. Distinct Effects of the Cervicovaginal Microbiota and Herpes Simplex Type 2 Infection on Female Genital Tract Immunology. J Infect Dis (2017) 215(9):1366–75. doi: 10.1093/infdis/jix088 PMC5451606 28201724

[57] Rizzo A Fiorentino M Buommino E Donnarumma G Losacco A Bevilacqua N. Lactobacillus Crispatus Mediates Anti-Inflammatory Cytokine Interleukin-10 Induction in Response to Chlamydia Trachomatis Infection *In Vitro* . Int J Med Microbiol (2015) 305(8):815–27. doi: 10.1016/j.ijmm.2015.07.005 26372530

[58] Chetwin E Manhanzva MT Abrahams AG Froissart R Gamieldien H Jaspan H. Antimicrobial and Inflammatory Properties of South African Clinical Lactobacillus Isolates and Vaginal Probiotics. Sci Rep (2019) 9(1):1917. doi: 10.1038/s41598-018-38253-4 30760770 PMC6374385

