# Supplementary material for: Temporal Changes in Vaginal Microbiota and Genital Tract Cytokines Among South African Women Treated for Bacterial Vaginosis
Source: Front Immunol. 2021 Sep 14;12:730986. doi: 10.3389/fimmu.2021.730986 (PMC8477043; doi:10.3389/fimmu.2021.730986)
Supplement: Supplementary file 1 [file Presentation_1.pptx]

## Slide 1
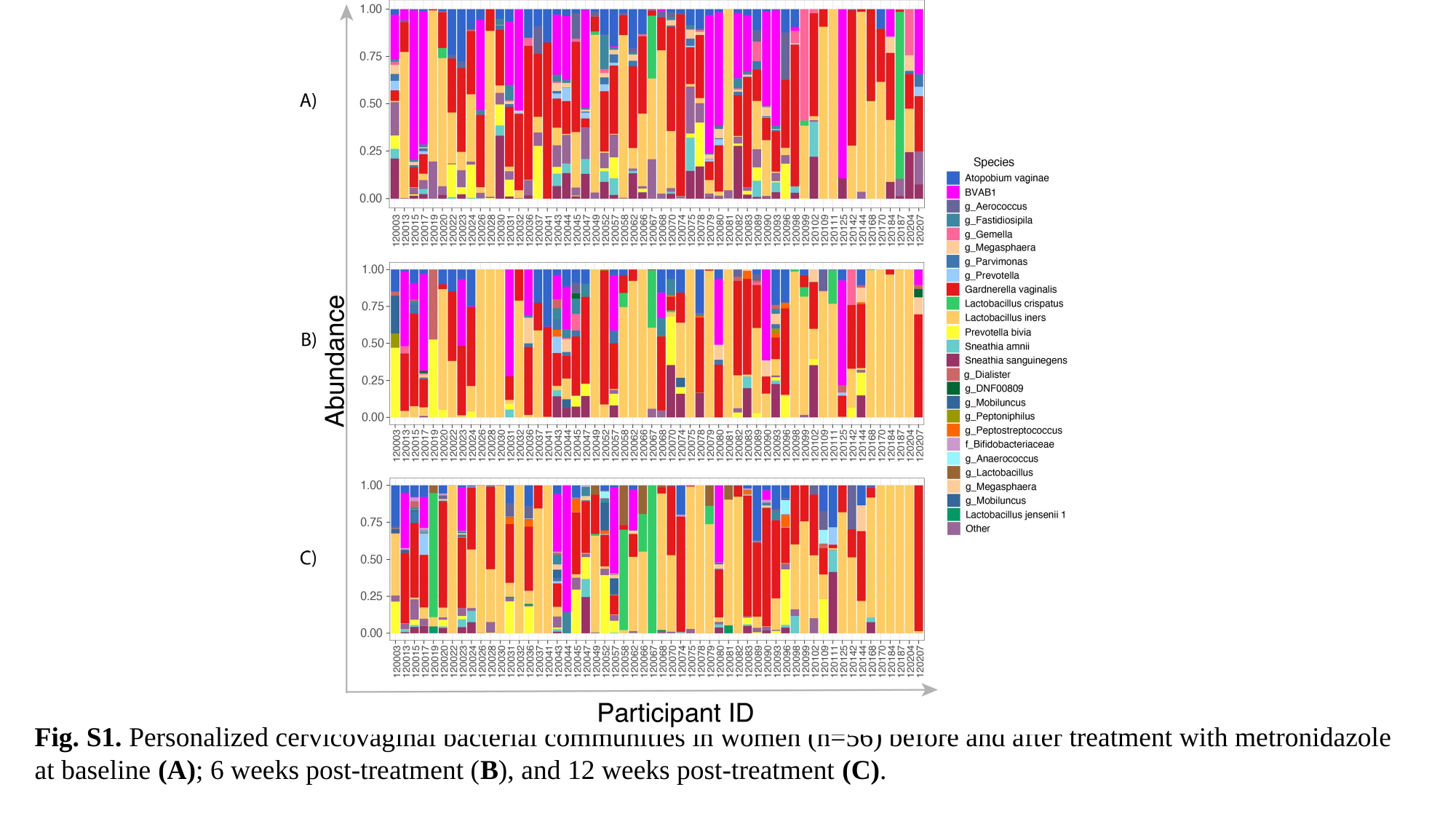

Fig. S1. Personalized cervicovaginal bacterial communities in women (n=56) before and after treatment with metronidazole at baseline (A); 6 weeks post-treatment (B), and 12 weeks post-treatment (C).

## Slide 2
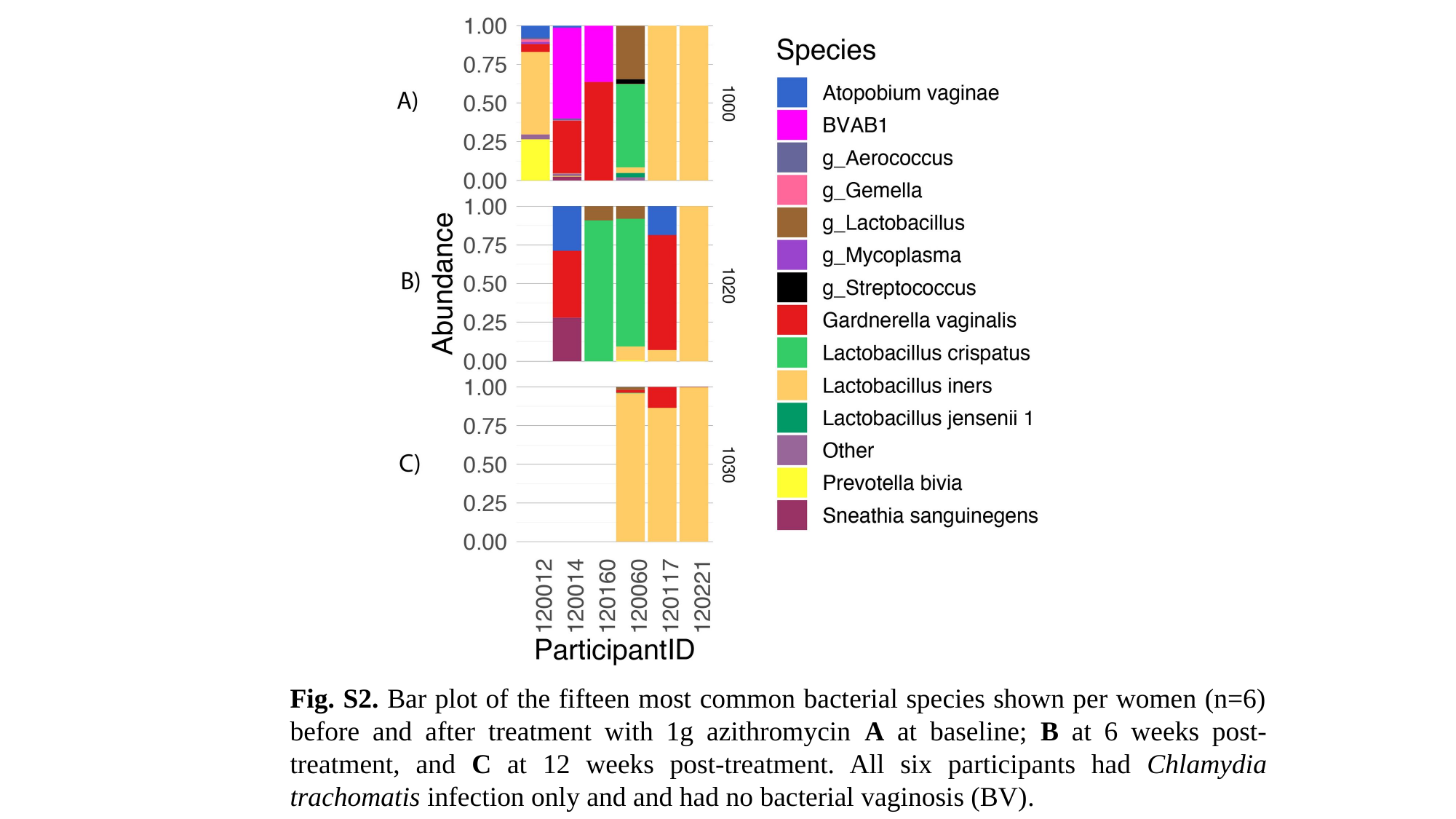

Fig. S2. Bar plot of the fifteen most common bacterial species shown per women (n=6) before and after treatment with 1g azithromycin A at baseline; B at 6 weeks post-treatment, and C at 12 weeks post-treatment. All six participants had Chlamydia trachomatis infection only and and had no bacterial vaginosis (BV).

## Slide 3
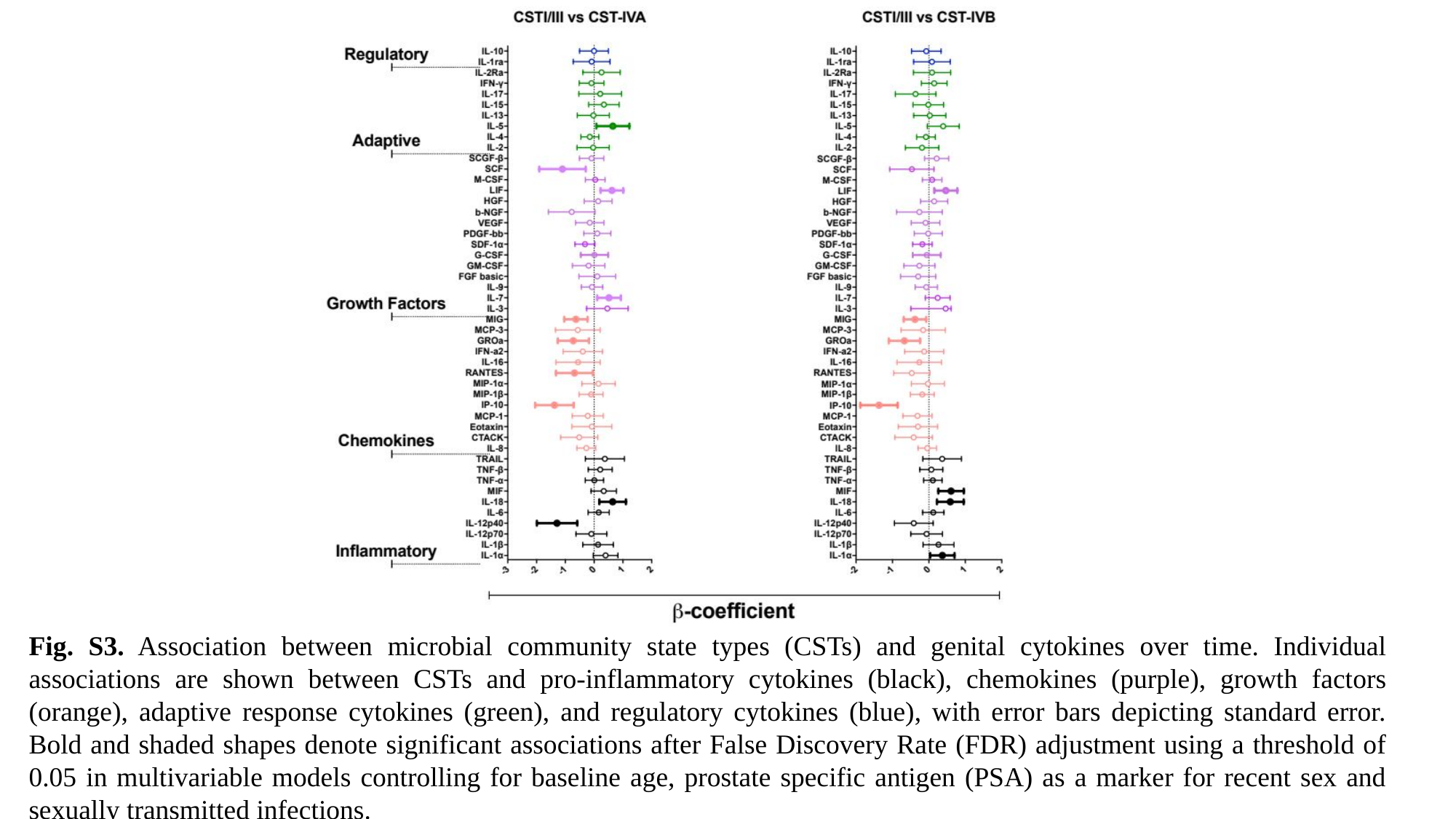

Fig. S3. Association between microbial community state types (CSTs) and genital cytokines over time. Individual associations are shown between CSTs and pro-inflammatory cytokines (black), chemokines (purple), growth factors (orange), adaptive response cytokines (green), and regulatory cytokines (blue), with error bars depicting standard error. Bold and shaded shapes denote significant associations after False Discovery Rate (FDR) adjustment using a threshold of 0.05 in multivariable models controlling for baseline age, prostate specific antigen (PSA) as a marker for recent sex and sexually transmitted infections.

## Slide 4
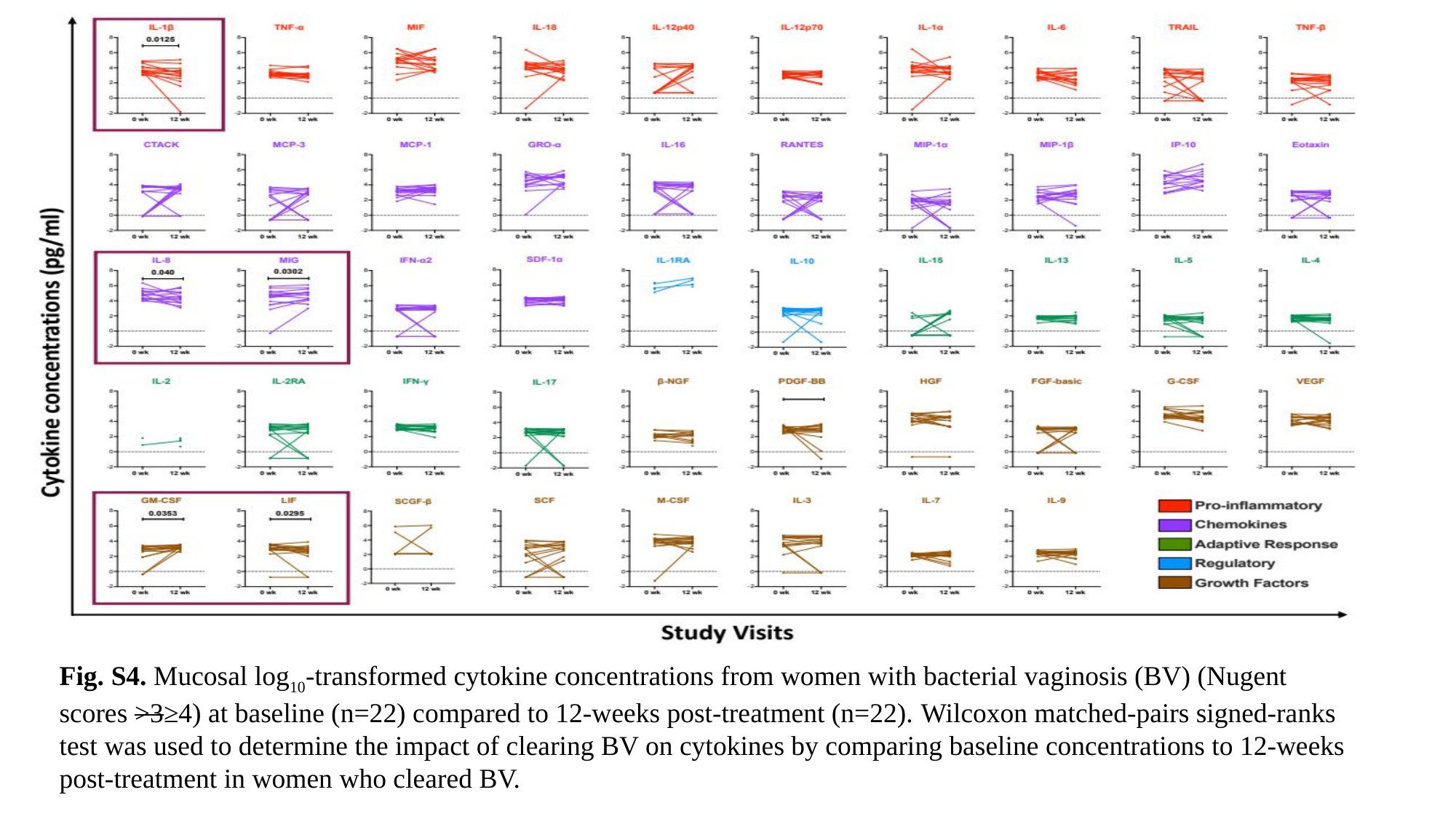

Fig. S4. Mucosal log10-transformed cytokine concentrations from women with bacterial vaginosis (BV) (Nugent scores >3≥4) at baseline (n=22) compared to 12-weeks post-treatment (n=22). Wilcoxon matched-pairs signed-ranks test was used to determine the impact of clearing BV on cytokines by comparing baseline concentrations to 12-weeks post-treatment in women who cleared BV.
