# Supplementary material for: Temporal Changes in Vaginal Microbiota and Genital Tract Cytokines Among South African Women Treated for Bacterial Vaginosis
Source: Front Immunol. 2021 Sep 14;12:730986. doi: 10.3389/fimmu.2021.730986 (PMC8477043; doi:10.3389/fimmu.2021.730986)
Supplement: Supplementary file 2 [file Table_1.docx]

**Supplementary Table 1A. Transitions between CSTs between baseline and follow up visits**

| **Weeks after treatment** | **CSTs** |  | **Intra-individual transitions across CSTs** | | | | | **P-value** |
| --- | --- | --- | --- | --- | --- | --- | --- | --- |
|  |  | **Overall N** | **I** | **III** | **IV-A** | **IV-B** | **IV-C** |  |
| **Baseline** | **I** | 1 | - | **-** | **-** | **-** | **-** | NA |
|  | **III** | 15 | **-** | - | **-** | **-** | **-** |  |
|  | **IV-A** | 17 | **-** | **-** | - | **-** | **-** |  |
|  | **IV-B** | 23 | **-** | **-** | **-** | - | **-** |  |
|  | **IV-C** | 0 | **-** | **-** | **-** | **-** | - |  |
| **Week 6** | **I** | 1 | 0 | 1 | 0 | 0 | 0 | >0.05 |
|  | **III** | 15 | 2 | 10 | 1 | 2 | 0 |  |
|  | **IV-A** | 16 | 0 | 3 | 7 | 6 | 1 |  |
|  | **IV-B** | 23 | 0 | 8 | 3 | 12 | 0 |  |
|  | **IV-C** | 0 | 0 | 0 | 0 | 0 | 0 |  |
| **Week 12** | **I** | 2 | 2 | 0 | 0 | 0 | 0 | >0.05 |
|  | **III** | 22 | 1 | 15 | 0 | 6 | 0 |  |
|  | **IV-A** | 11 | 0 | 1 | 7 | 3 | 0 |  |
|  | **IV-B** | 20 | 0 | 9 | 0 | 11 | 0 |  |
|  | **IV-C** | 1 | 0 | 1 | 0 | 0 | 0 |  |

*P values calculated using an omnibus symmetry exact test for a paired contingency table, CST: community state type.*

**Supplementary Table 1B. Association of CSTs between baseline and follow up visits**

| **CSTs** | **I** | **III** | **IV-A** | **IV-B** | **IV-C** |
| --- | --- | --- | --- | --- | --- |
| **I** | **-** | 1 | N/A | N/A | N/A |
| **III** | 1 | - | 1 | 0.607 | 1 |
| **IV-A** | N/A | 0.625 | - | 1 | N/A |
| **IV-B** | N/A | 0.109 | 0.508 | - | N/A |
| **IV-C** | N/A | N/A | 1 | N/A | - |

*Comparison between community state types (CSTs) at 6 and 12 weeks after treatment are shown in green ad grey colours, respectively. P values calculated using pairwise symmetry tests.*

Supplementary Table 2: Relationship between CSTs and genital tract cytokines

| **Cytokine** | **CST-I/III** | **CST-IVA** | | **P-value** |  | **CST-IVB** | **P-value** |
| --- | --- | --- | --- | --- | --- | --- | --- |
|  | Median (IQR) | Median (IQR) | β-coefficient (IQR) |  | Median (IQR) | β-coefficient (IQR) |  |
|  |  |  |  |  |  |  |  |
| **b-NGF** | 1.58 (0.76 - 2.29) | 1.6 (0.69 - 2.12) | -0.77 (-1.58 - 0.03) | 0.060 | 1.67 (1.11 - 2.12) | -0.26 (-0.89 - 0.37) | 0.416 |
| **CTACK** | 3.55 (3.27 - 3.74) | 3.35 (2.87 - 3.62) | -0.52 (-1.16 - 0.13) | 0.116 | 3.43 (2.51 - 3.66) | -0.42 (-0.93 - 0.09) | 0.110 |
| **Eotaxin** | 2.4 (-0.35 - 2.88) | 2.22 (-0.35 - 2.73) | -0.08 (-0.77 - 0.62) | 0.828 | 2.04 (-0.35 - 2.88) | -0.3 (-0.84 - 0.24) | 0.274 |
| **FGF basic** | 2.75 (2.21 - 3.06) | 2.66 (-0.17 - 2.97) | 0.11 (-0.53 - 0.75) | 0.733 | 2.74 (-0.17 - 3.04) | -0.29 (-0.78 - 0.19) | 0.233 |
| **G-CSF** | 4.26 (3.82 - 4.88) | 4.26 (3.91 - 4.84) | 0.01 (-0.46 - 0.49) | 0.957 | 4.34 (3.84 - 4.8) | -0.06 (-0.44 - 0.33) | 0.770 |
| **GM-CSF** | 3.16 (2.92 - 3.3) | 3.07 (2.86 - 3.22) | -0.19 (-0.75 - 0.37) | 0.510 | 3.09 (2.7 - 3.25) | -0.26 (-0.68 - 0.17) | 0.233 |
| **GRO-a** | 4.88 (4.03 - 5.33) | 4.23 (3.56 - 5.05) | -0.72 (-1.26 - -0.18) | **0.009** | 4.61 (3.56 - 5.08) | -0.67 (-1.1 – (-0.24)) | **0.002** |
| **HGF** | 3.92 (3.3 - 4.5) | 3.99 (3.47 - 4.46) | 0.14 (-0.34 - 0.63) | 0.567 | 4.11 (3.53 - 4.47) | 0.15 (-0.23 - 0.52) | 0.445 |
| **IFN-a2** | 2.67 (1.49 - 3.12) | 2.63 (-0.71 - 2.92) | -0.39 (-1.08 - 0.29) | 0.259 | 2.72 (0.36 - 3) | -0.13 (-0.67 - 0.41) | 0.637 |
| **IFN-γ** | 2.98 (2.71 - 3.3) | 2.92 (2.66 - 3.23) | -0.09 (-0.52 - 0.34) | 0.686 | 3.09 (2.8 - 3.39) | 0.15 (-0.21 - 0.5) | 0.417 |
| **IL-10** | 2.82 (2.39 - 3.02) | 2.71 (2.3 - 2.95) | 0 (-0.51 - 0.5) | 0.988 | 2.86 (2.38 - 3.1) | -0.07 (-0.48 - 0.33) | 0.729 |
| **IL-12p70** | 3.07 (2.52 - 3.36) | 2.98 (2.51 - 3.27) | -0.09 (-0.63 - 0.44) | 0.728 | 3.19 (2.56 - 3.4) | -0.06 (-0.5 - 0.37) | 0.782 |
| **IL-12p40** | 3.23 (0.71 - 4.21) | 0.71 (0.71 - 3.56) | -1.29 (-1.99 - -0.59) | **0.000** | 0.71 (0.71 - 3.92) | -0.41 (-0.95 - 0.12) | 0.126 |
| **IL-13** | 1.63 (0.95 - 1.9) | 1.55 (0.98 - 1.81) | -0.03 (-0.59 - 0.53) | 0.920 | 1.69 (1.29 - 1.93) | 0.03 (-0.42 - 0.47) | 0.908 |
| **IL-15** | -0.56 (-0.56 - 0.27) | -0.56 (-0.56 - 1.44) | 0.34 (-0.19 - 0.87) | 0.207 | -0.56 (-0.56 – (-0.56)) | -0.02 (-0.43 - 0.4) | 0.941 |
| **IL-16** | 3.22 (0.16 - 3.87) | 3.15 (0.16 - 3.83) | -0.56 (-1.33 - 0.21) | 0.155 | 3.15 (0.16 - 4.03) | -0.26 (-0.87 - 0.35) | 0.401 |
| **IL-17** | 2.5 (2.04 - 2.92) | 2.38 (1.78 - 2.82) | 0.21 (-0.53 - 0.95) | 0.574 | 2.39 (1.78 - 2.7) | -0.36 (-0.92 - 0.19) | 0.199 |
| **IL-18** | 3.65 (3.04 - 4.05) | 4.01 (3.39 - 4.62) | 0.64 (0.18 - 1.1) | **0.006** | 4.08 (3.66 - 4.53) | 0.59 (0.22 - 0.96) | **0.002** |
| **IL-1α** | 3.55 (3.16 - 4.17) | 3.84 (3.34 - 4.4) | 0.4 (-0.03 - 0.83) | 0.070 | 3.93 (3.54 - 4.38) | 0.37 (0.04 - 0.7) | **0.030** |
| **IL-1β** | 3.27 (2.71 - 3.82) | 3.37 (2.79 - 3.75) | 0.14 (-0.39 - 0.67) | 0.614 | 3.48 (3.01 - 4.16) | 0.27 (-0.16 - 0.69) | 0.22 |
| **IL-1ra** | 8.43 (6.16 - 8.43) | 8.43 (5.7 - 8.43) | -0.08 (-0.72 - 0.55) | 0.796 | 8.43 (6.29 - 8.43) | 0.09 (-0.42 - 0.59) | 0.738 |
| **IL-2** | -2.3 (-2.3 - -2.3) | -2.3 (-2.3 - (-2.3)) | -0.03 (-0.59 - 0.53) | 0.906 | -2.3 (-2.3 – (-2.3)) | -0.19 (-0.65 - 0.27) | 0.425 |
| **IL-2Ra** | 2.55 (1.5 - 3.27) | 2.7 (1.98 - 3.11) | 0.26 (-0.39 - 0.91) | 0.433 | 2.68 (2.17 - 3.18) | 0.09 (-0.42 - 0.6) | 0.737 |
| **IL-3** | 3.6 (2.71 - 4.46) | 3.63 (2.97 - 4.42) | 0.46 (-0.26 - 1.18) | 0.210 | 3.59 (2.76 - 4.47) | 0.46 (-0.49 - 0.61) | 0.838 |
| **IL-4** | 1.53 (1.23 - 1.76) | 1.44 (1.18 - 1.62) | -0.15 (-0.46 - 0.16) | 0.350 | 1.5 (1.31 - 1.79) | -0.08 (-0.34 - 0.18) | 0.537 |
| **IL-5** | 1.27 (-0.74 - 1.69) | 1.04 (-0.74 - 2.03) | 0.65 (0.08 - 1.22) | **0.026** | 1.6 (-0.74 - 1.98) | 0.39 (-0.04 - 0.83) | 0.078 |
| **IL-6** | 2.44 (2.01 - 2.98) | 2.55 (2.03 - 3.03) | 0.16 (-0.21 - 0.52) | 0.399 | 2.67 (2.37 - 3.09) | 0.12 (-0.17 - 0.41) | 0.416 |
| **IL-7** | 2.05 (1.53 - 2.33) | 2.15 (1.65 - 2.36) | 0.52 (0.11 - 0.93) | **0.013** | 2.1 (1.82 - 2.36) | 0.24 (-0.1 - 0.58) | 0.159 |
| **IL.8** | 4.47 (4.02 - 4.99) | 4.26 (3.87 - 4.73) | -0.27 (-0.6 - 0.06) | 0.105 | 4.54 (4.06 - 5.16) | -0.04 (-0.29 - 0.21) | 0.74 |
| **IL-9** | 2.33 (2.08 - 2.52) | 2.28 (2.07 - 2.46) | -0.07 (-0.44 - 0.3) | 0.702 | 2.29 (2.04 - 2.53) | -0.07 (-0.38 - 0.23) | 0.648 |
| **IP-10** | 4.45 (3.85 - 5.55) | 3.76 (3.09 - 4.57) | -1.38 (-2.05 –(-0.71)) | **0.000** | 3.99 (2.75 - 4.78) | -1.37 (-1.88 – (-0.86)) | **0.000** |
| **LIF** | 2.61 (2.22 - 3.1) | 2.9 (2.38 - 3.3) | 0.62 (0.23 - 1.01) | **0.002** | 2.9 (2.5 - 3.42) | 0.47 (0.15 - 0.78) | **0.004** |
| **M-CSF** | 3.7 (3.33 - 4.13) | 3.69 (3.35 - 4.13) | 0.04 (-0.31 - 0.38) | 0.829 | 3.73 (3.32 - 4.13) | 0.09 (-0.18 - 0.36) | 0.509 |
| **MCP-1** | 3.14 (2.84 - 3.43) | 2.99 (2.56 - 3.24) | -0.22 (-0.76 - 0.32) | 0.421 | 2.95 (2.38 - 3.24) | -0.31 (-0.71 - 0.09) | 0.127 |
| **MCP-3** | 1.44 (-0.63 - 3.17) | -0.63 (-0.63 - 2.67) | -0.57 (-1.34 - 0.21) | 0.154 | 1.62 (-0.63 - 2.89) | -0.16 (-0.76 - 0.45) | 0.614 |
| **MIF** | 4.54 (3.77 - 5.08) | 4.89 (3.87 - 5.28) | 0.33 (-0.11 - 0.77) | 0.137 | 5.1 (4.66 - 5.5) | 0.61 (0.26 - 0.96) | **0.001** |
| **MIG** | 4.48 (3.97 - 5.12) | 4.06 (3.64 - 4.69) | -0.63 (-1.04 - -0.23) | **0.002** | 4.38 (3.54 - 5.06) | -0.38 (-0.69 – (-0.07)) | **0.016** |
| **MIP-1α** | 1.65 (1.05 - 2.01) | 1.61 (1.23 - 1.92) | 0.15 (-0.42 - 0.73) | 0.601 | 1.67 (1.2 - 1.99) | -0.03 (-0.48 - 0.43) | 0.905 |
| **MIP-1β** | 2.44 (1.87 - 3) | 2.37 (1.72 - 2.78) | -0.11 (-0.52 - 0.31) | 0.619 | 2.32 (1.87 - 2.68) | -0.18 (-0.51 - 0.14) | 0.276 |
| **PDGF-bb** | 2.68 (2.27 - 3.11) | 2.69 (2.23 - 3.08) | 0.11 (-0.36 - 0.58) | 0.641 | 2.69 (2.28 - 3) | -0.02 (-0.4 - 0.37) | 0.932 |
| **RANTES** | 1.98 (-0.52 - 2.49) | -0.52 (-0.52 - 2.3) | -0.68 (-1.32 - -0.04) | **0.037** | 1.35 (-0.52 - 2.37) | -0.47 (-0.96 - 0.03) | 0.065 |
| **SCF** | 2.42 (-0.76 - 3.45) | 1.17 (-0.76 - 2.93) | -1.1 (-1.91 – (-0.29)) | **0.008** | 2.29 (-0.76 - 3.23) | -0.47 (1.07 - 0.14) | 0.132 |
| **SCGF-β** | 2.07 (2.07 - 2.07) | 2.07 (2.07 - 2.07) | -0.09 (-0.51 - 0.34) | 0.693 | 2.07 (2.07 - 2.07) | 0.21 (-0.12 - 0.55) | 0.205 |
| **SDF-1α** | 3.99 (3.5 - 4.18) | 3.79 (3.46 - 4.11) | -0.32 (-0.66 - 0.03) | 0.072 | 3.91 (3.53 - 4.19) | -0.18 (-0.45 - 0.09) | 0.191 |
| **TNF-α** | 2.93 (2.71 - 3.29) | 2.95 (2.71 - 3.27) | 0.01 (-0.31 - 0.33) | 0.957 | 3.15 (2.8 - 3.42) | 0.11 (-0.14 - 0.36) | 0.392 |
| **TNF-β** | 2.05 (1.56 - 2.64) | 2.17 (1.71 - 2.5) | 0.21 (-0.2 - 0.63) | 0.319 | 2.23 (1.71 - 2.64) | 0.07 (-0.25 - 0.38) | 0.678 |
| **TRAIL** | 2.46 (-0.37 - 3.25) | 2.61 (1.44 - 3.26) | 0.37 (-0.31 - 1.05) | 0.281 | 2.75 (1.08 - 3.47) | 0.36 (-0.16 - 0.89) | 0.176 |
| **VEGF** | 4.13 (3.53 - 4.62) | 4.04 (3.52 - 4.43) | -0.15 (-0.65 - 0.34) | 0.549 | 4.27 (3.59 - 4.59) | -0.09 (-0.48 - 0.3) | 0.648 |

Supplementary Table 3: Impact of metronidazole treatment on temporal dynamics of mucosal cytokines profiles

**
